# Balanced gene expression of homeologs suggests strong dosage sensitivity and gene longevity after paleopolyploidies in angiosperms

**DOI:** 10.1093/plcell/koae227

**Published:** 2024-10-03

**Authors:** Tao Shi, Zhiyan Gao, Jinming Chen, Yves Van de Peer

**Affiliations:** 1 Aquatic Plant Research Center, Wuhan Botanical Garden, Chinese Academy of Sciences, Wuhan 430074, China; 2 Department of Plant Biotechnology and Bioinformatics, Ghent University, Ghent, Belgium; 3 Centre for Plant Systems Biology, VIB, Ghent, Belgium; 4 Department of Biochemistry, Genetics and Microbiology, University of Pretoria, 0028 Pretoria, South-Africa; 5 College of Horticulture, Academy for Advanced Interdisciplinary Studies, Nanjing Agricultural University, 210095 Nanjing, China

**Keywords:** whole-genome duplication (WGD), balanced expression, dosage-balance constraint, gene retention, angiosperms

## Abstract

Following whole-genome duplication (WGD), duplicate gene pairs (homeologs) can evolve varying degrees of expression divergence. However, the determinants influencing these relative expression level differences (*R*_FPKM_) between homeologs remain elusive. Here, we analyzed the *R*_FPKM_ between homeologs in three angiosperms, *Nymphaea, Nelumbo*, and *Acorus*, all having undergone a single WGD since the origin of angiosperms. Our results show significant positive correlations in *R*_FPKM_ of homeologs among tissues within the same species, and among orthologs across these three species, indicating convergent expression balance/bias between homeologous gene copies following independent WGDs. We linked *R*_FPKM_ between homeologs to gene attributes associated with dosage balance constraints, such as protein-protein interactions, lethal-phenotype scores in Arabidopsis orthologs, domain numbers, and expression breadth. Notably, homeologs with lower *R*_FPKM_ often had more interactions and higher lethal-phenotype scores, indicating selective pressures favoring balanced expression. Also, homeologs with lower *R*_FPKM_ were more likely to be retained after WGDs in angiosperms. Within *Nelumbo*, greater *R*_FPKM_ between homeologs correlated with increased *cis*- and *trans*-regulatory differentiation between species, highlighting the ongoing escalation of gene expression divergence. We further found that expression degeneration in one copy of homeologs is inclined towards nonfunctionalization. Our research highlights the importance of balanced expression, shaped by dosage balance constraints, in the evolutionary retention of homeologs in plants.

## Introduction

Gene duplication provides extra genetic material for evolution to work on (Ohno, 1970). Polyploidy, resulting from whole-genome duplication (WGD) where the entire set of genes is duplicated simultaneously, has been assumed to facilitate species diversification and survival under environmental turmoil (Lynch and Conery, 2000; Lynch and Force, 2000; Van de Peer et al., 2017; Fox et al., 2020; Roman-Palacios et al., 2020; Wu et al., 2020; Van de Peer et al., 2021; Ebadi et al., 2023). Although the most likely fate of duplicated genes is gene loss (Lynch and Conery, 2000), even after tens to hundreds of millions of years, a substantial portion of the homeologs (genes duplicated in a WGD event) remain present in the extant genomes. The retention of both copies is often explained by assuming subfunctionalization (Force et al., 1999) or neofunctionalization (Ohno, 1970) of genes, by gene dosage effects (Conant and Wolfe, 2008) or dosage-balance constraints (Birchler and Veitia, 2007), or other mechanisms (Van de Peer et al., 2017), including the preferential retention of biologically meaningful gene clusters of interacting genes (i.e., genes encoding proteins acting in multiprotein complexes), including those with coexpression or preservation of epistatic interactions (Makino and McLysaght, 2012). Of all theories of WGD-derived duplicate gene trajectories (deletion vs. retention), dosage sensitivity or dosage balance constraint effects seems particularly important, as directly impacting gene dispensability and duplicability (Tasdighian et al., 2017; Birchler and Yang, 2022). The dosage balance hypothesis asserts that selection acts on the expressed amount of gene product to maintain the stoichiometry of protein complex subunits, crucial for their proper functioning (Papp et al., 2003; Birchler and Yang, 2022). For polyploids, WGD often triggers gene loss and genomic reshuffling, yet many duplicates escape deletion due to such dosage balance constraints to maintain proper stoichiometric balance, contributing greatly to the specific gene content of paleopolyploids or younger mesopolyploids (Geiser et al., 2016; Cheng et al., 2018; Kuzmin et al., 2020). Evidence in angiosperms also indicates that genes that function in 'development' and 'transcription regulation', likely sensitive to dosage balance, are typically retained as duplicates post- WGD for millions of years while others eventually revert to single-copy status in most species (Maere et al., 2005; Li et al., 2016).

The fate of a redundant gene copy is indeed largely subjected to dosage-balance constraints because it is the amount of gene product (protein) that is firstly ‘visible’ for natural selection. Although both regulatory and protein-coding regions are being duplicated through WGD, gene expression, a critical determinant of gene function for protein coding genes, often exhibits substantial divergence in duplicate gene copies over time. A previous study in *Arabidopsis* revealed that small-scale duplicates exhibit a more pronounced asymmetry in expression divergence between copies compared to duplicates derived from large-scale segmental duplications or WGDs (Casneuf et al., 2006). Yet, also duplicates (homeologs) from WGDs can show substantial divergence in expression. This divergence often manifests itself as one copy dominating in expression (the other copy then shows ‘expression degeneration') across different tissues, as frequently observed in various species, such as the common carp (Li et al., 2015) and different plant species (Ganko et al., 2007; Liang and Schnable, 2018). However, there are instances where a fraction of homeologs retain ‘balanced’ expression levels, as evidenced in *Arabidopsis* (Coate et al., 2020). In another example, the *Petunia hybrida* homeologs *PhGLO1* and *PhGLO2*, both B-class MADS box proteins, maintain similar expression levels likely due to functional constraints from the formation of heterodimers like PHDEF/PHGLO1 and PHDEF/PHGLO2, crucial for their redundant roles in flower development (Vandenbussche et al., 2004). The potential range of expression level variation of copies among different homeologous pairs, from dominance to balanced expression, does not seem to be random but indicates a complex expression landscape, where dosage balance constraints might play a significant role as stoichiometry of interacting proteins is maintained through intricate gene expression and synthesis of the proper amounts of proteins.

The intriguing patterns of gene expression level divergence between homeologous copies after a WGD, and particularly what may constrain it, needs further investigation. By focusing on plants that have undergone only one single WGD since the origin of angiosperms, such as *Nelumbo* (a sister lineage of most other eudicots), *Acorus* (a sister lineage of most other monocots), and *Nymphaea* (a member of the ANA-grade, a sister lineage of most other angiosperms including eudicots, monocots, magnoliids and others)(Ming et al., 2013; Zhang et al., 2019; Shi and Chen, 2020; Shi et al., 2020; Shi et al., 2022), we can eliminate confounding effects of recurring duplications (at least through WGD) within the same organism (Tiley et al., 2016). As this setting assures a uniform genesis for all WGD-derived duplicates per species, differences in expression between copies of homeologs must be due to their differences in regulatory evolution rates. In this study, we aim to investigate the multiple factors associated with dosage balance constraints that may hypothetically limit the expression divergence of the duplicate copy, such as protein-protein interactions, the number of protein domains encoded in genes, protein length, expression breadth in various tissues, and so on. Our primary goal is to elucidate the transition from balanced to unbalanced (or not) gene expression between gene copies in plant species characterized by a single WGD. Further, given the availability of data including *cis*- and *trans*-regulatory changes between two *Nelumbo* species (Li et al., 2021a; Li et al., 2021b; Zhang et al., 2022b; Gao et al., 2023), we aim to assess the dosage balance constraint on the ongoing and escalating evolutionary differences between duplicate gene pairs with varying degrees of expression bias during the last ~6.5 million years. Finally, we analyze the evolutionary, structural, and functional traits of high-expression versus low-expression homeologous copies to gain insights into how differences in expression levels between copies may manifest divergent evolutionary outcomes.

## Results

### Convergence of expression divergence of homeologs

**Figure 1:**
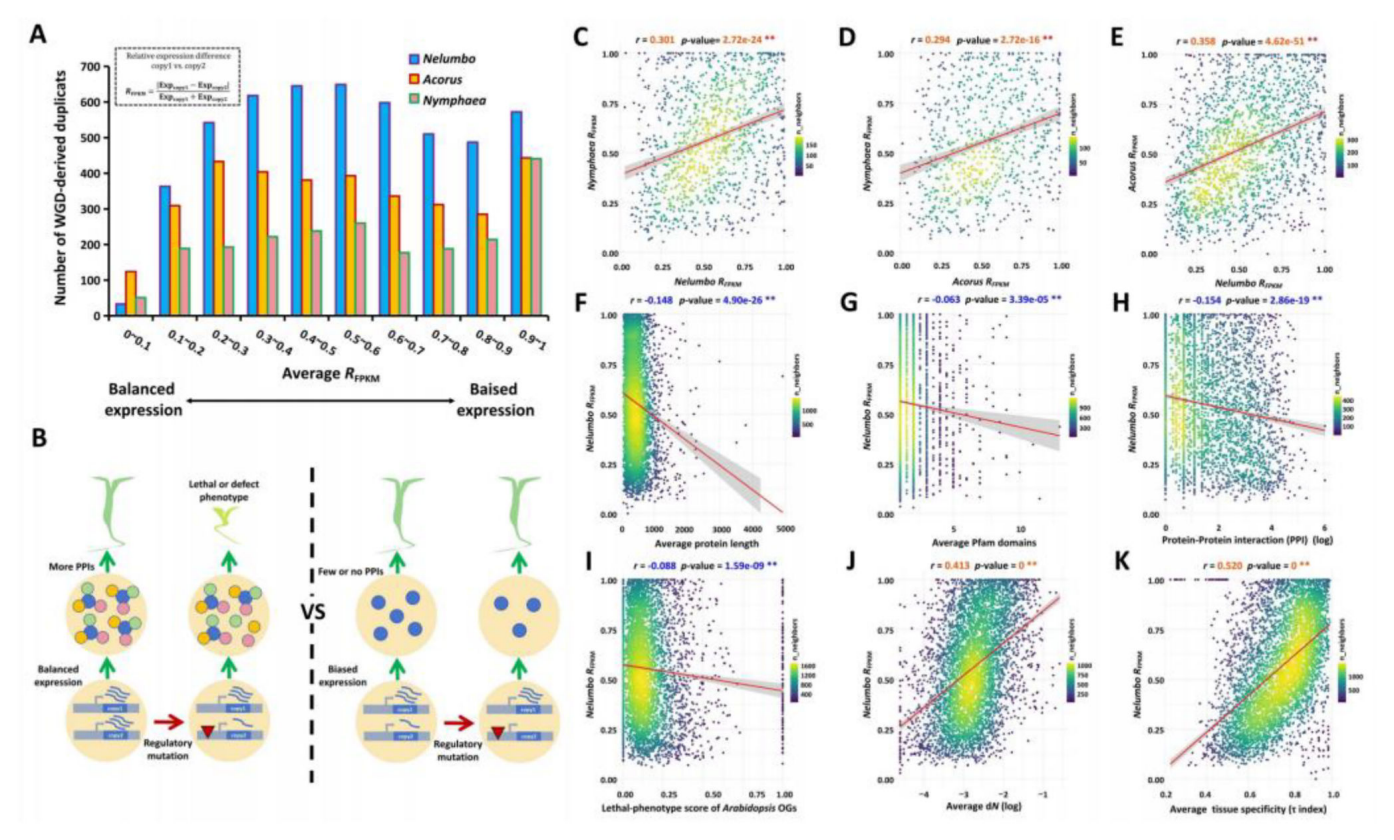
Gene expression characteristics of homeologs for *Nelumbo nucifera, Acorus tatarinowii*, and *Nymphaea colorata*. **A**. Distributions of the proportion of homeologs according to relative expression difference between the two homeologous copies (*R*_FPKM_) for *Nymphaea, Nelumbo* and *Acorus*. **B**. Hypothesis showing that higher expression balance of duplicate copies is associated with more protein-protein interactions and higher sensitivity to expression change. **C-E**. Average *R*_FPKM_ of orthologous duplicates are significantly correlated between *Nymphaea* and *Nelumbo* (**C**), *Nymphaea* and *Acorus* (**D**), and *Nelumbo* and *Acorus* (**E**). **F-K**. Average *R*_FPKM_ of WGD duplicates in *Nelumbo* are significantly negatively correlated with average protein length (**F**), No. of Pfam domains genes (**G**), No. of protein-protein interactions of orthologs in *Arabidopsis* (**H**), lethal-phenotype score of *Arabidopsis* ortholog groups (**I**), average non-synonymous substitutions per site after WGD (**J**), and significantly positively correlated with tissue specificity (τ) of gene expression (**K**). *r*, correlation coefficient of Pearson correlation test; **, *p*-value < 0.01; log, log-transformed values of gene features.

Chromosome-level genome assemblies of *Nymphaea colorata, Nelumbo nucifera*, and *Acorus tatarinowii*, achieving high BUSCO scores of 94.40%, 94.60%, and 92.40%, respectively, have previously been published (Supplementary Table S1). These well-assembled and well-annotated genomes ensure high-quality datasets for our subsequent analyses. After eliminating redundant homeologous gene pairs associated with tandem arrays (see Materials and Methods), we obtained 2,442, 5,018, and 3,631 homeologous gene pairs through intra-specific synteny searches from the 31,475, 34,233, and 28,241 annotated genes in *Nymphaea, Nelumbo*, and *Acorus*, respectively (Supplementary Table S2). First, to uncover how different homeologs in *Nymphaea, Nelumbo*, and *Acorus* may differ in expression, we summarized the relative frequency distribution of homeologs according to their expression level difference (Figure 1A). In total, 19, 11, and 5 tissues were used to measure expression level differences between homeologous copies for *Nelumbo, Nymphaea*, and *Acorus*, respectively (Supplementary Table S3). To ensure comparability of gene expression divergence or expression decay of one of the homeologs across species and across homeologous gene pairs, we normalized the expression differences between duplicate copies as *R*_FPKM_ for each tissue sample (see Materials and Methods and Figure 1A). *R*_FPKM_ values range from 0 to 1, i.e., from no difference (no divergence) in gene expression between both duplicates to complete silence of one copy (Figure 1A). The *R*_FPKM_ of homeologs within the same species shows a high and significant correlation across different tissues, as indicated by Pearson correlation tests (all *p*-values < 0.01), suggesting that the extent of gene expression divergence among homeologs is highly consistent and stable across various plant tissues (Supplementary Figure S1, Supplementary Table S4). Consequently, our subsequent analyses of homeolog expression evolution primarily utilize the average *R*_FPKM_ values across all tissues. Generally, in all three species studied, the distribution of homeologs is skewed towards higher *R*_FPKM_ values (Figure 1A). This trend indicates that for most duplicate pairs, one copy undergoes considerable expression degeneration (referred to as biased expression) following WGD, while a subset maintains balanced expression levels between the copies.

Over tens of millions of years following a single WGD, it remains noteworthy that a fraction of homeologs persist with highly balanced (highly similar) expression levels. This observation suggests the presence of a stringent selective pressure, likely driven by the critical dosage balance required for protein-protein interactions. Such selective constraints are essential to mitigate expression alterations and to ensure the production of the correct number of interacting proteins in multiprotein complexes within the cell, thus preventing dysfunctional or lethal phenotypes (Figure 1B). Should such constraints on gene expression last for extended periods, we would anticipate that orthologous duplicates between species exhibit consistent patterns in expression balance or bias following independent WGDs in each species. In agreement with this hypothesis, we observed significant correlations in the average *R*_FPKM_ of orthologous duplicates between *Nymphaea* and *Nelumbo* (Figure 1C), *Nymphaea* and *Acorus* (Figure 1D), and *Nelumbo* and *Acorus* (Figure 1E). These correlations underscore the influence of selective pressures in sculpting the convergent patterns of expression balance or unbalance among orthologs across these species in the context of their respective WGDs.

**Figure 2:**
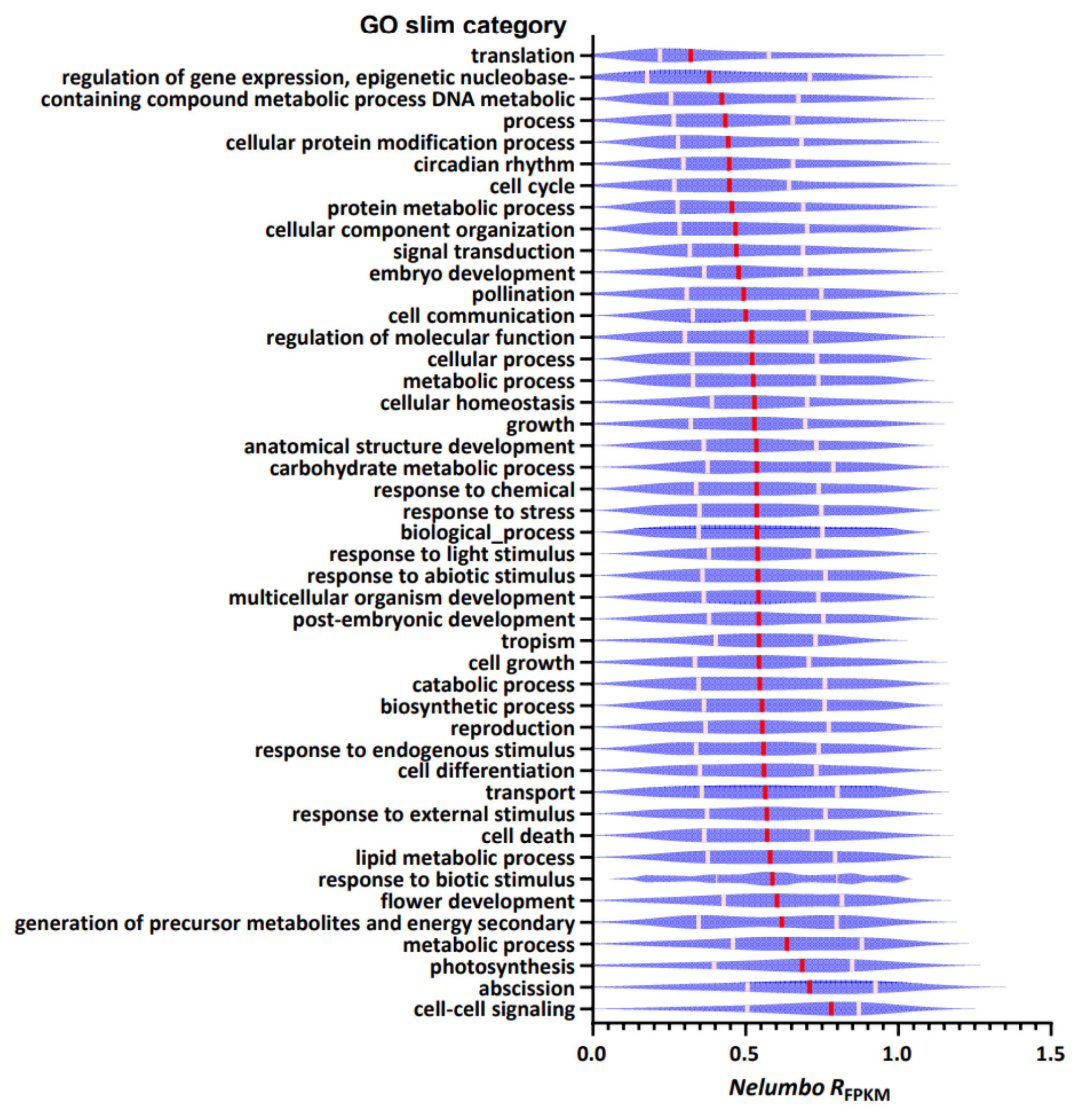
Violin plots illustrating relative expression difference (*R*_FPKM_) of *Nelumbo* homeologs varies among Gene Ontology (GO) slim categories. Red bar, median; pink bar, quartile.

To investigate whether, and to what extent, dosage balance constraints or negative selection influence the relative expression differences in homeologs across three species, we explored the relationships between the average *R*_FPKM_ of WGD duplicates and a range of gene characteristics, encompassing structural, functional, and molecular evolutionary aspects, with both linear and log-transformed regression analyses (Supplementary Table S5). Consistently, our findings reveal that, although the Pearson correlation coefficient suggests a weak correlation between *R*_FPKM_ and Pfam domain count (*r*<-0.1), the average *R*_FPKM_ of duplicates in all three species exhibits a significant negative correlation with several gene attributes: protein length, number of exons, Pfam domain count per gene, and the number of protein-protein interactions of orthologs in *Arabidopsis* (Pearson correlation tests, all *p*-values < 0.01) (Figure 1F-H, Table 1, Supplementary Figure S2, S3, S4). After transferring the lethal-phenotype scores from *Arabidopsis* (Lloyd et al., 2015) to our three investigated species via ortholog assignment, our analysis revealed that the average *R*_FPKM_ of duplicates across the three species displays a significantly negative correlation with the lethal-phenotype score of their orthologs in *Arabidopsis*, although the correlation coefficients are weak (*r* < -0.1) (Figure 1I, Supplementary Figure S3J, S4J)(all *p*-values < 0.01). This finding indicates a potential negative selection on these homeologs with balanced expression, as evidenced by Pearson correlation tests (Figure 1I, Supplementary Figure S3J, S4J). In line with this, we observed that homeologs exhibiting balanced expression typically demonstrate lower tissue-specificity (τ) in gene expression, implying their involvement in multiple plant tissue types and a higher degree of essentiality (Figure 1K, Supplementary Figure S3D, S4C). This conclusion is further supported by molecular evolutionary data, where average *R*_FPKM_ shows significant positive correlations with both synonymous (*dS*) and, notably, non-synonymous (*dN*) substitution rates (Pearson correlation tests, all *p*-value<0.01) (Figure 1J, Supplementary Figure S2A, S3A-B, S4A-B). The stronger correlation with *dN* is consistent with a previous finding in *Arabidopsis*, indicating rapid protein evolution associated with expression change (Ganko et al., 2007). Yet, *R*_FPKM_ only have significantly positive correlations with d*N*/d*S* ratio (*ω*) in *Nymphaea* and *Nelumbo* but not *Acorus*, possibly because of other selective pressures acting in this context (Supplementary Figure S2B, S3C, S4D). When we independently conducted correlation tests between the *R*_FPKM_ of homeologs for each individual plant tissue and a variety of gene characteristics, we observed correlation patterns similar to those found using the average *R*_FPKM_: while the p-values for individual tissues are slightly higher, the correlation coefficients (*r*) remain consistent between average *R*_FPKM_ and those of individual tissues (Supplementary Table S5). This suggests that increasing the number of tissues sampled for average *R*_FPKM_ can lead to lower p-values, but the overall trends in positive or negative correlations remain unchanged if we use different individual tissue samples. In addition, we compared correlation coefficients (*r*) of *R*_FPKM_ and gene traits among *Nymphaea, Nelumbo* and *Acorus* listed in Table 1, and we found convergent trends in *R*_FPKM_ -gene-trait relationships among species based on significant Pearson correlations (Supplementary Figure S5). Further, upon categorizing various homeologs into Gene Ontology (GO) slim categories, it was observed that those linked to dosage-sensitive and complex systems, notably in categories like ‘regulation of gene expression, epigenetic’ and ‘translation,’ tend to have some of lower average *R*_FPKM_ values. Conversely, homeologs involved in ‘secondary metabolic process’ and ‘response to biotic stimulus’ exhibit some of higher average *R*_FPKM_ values (Figure 2, Supplementary Figure S6, S7), supporting a previous study that stress-regulated genes evolve rapidly in expression (Zou et al., 2009). These functional annotations further corroborate the significance of dosage balance in constraining the expression level evolution between homeologs.

### Expression balance between homeologs predicts copy number change of angiosperm orthologs experiencing different rounds of WGDs

**Figure 3:**
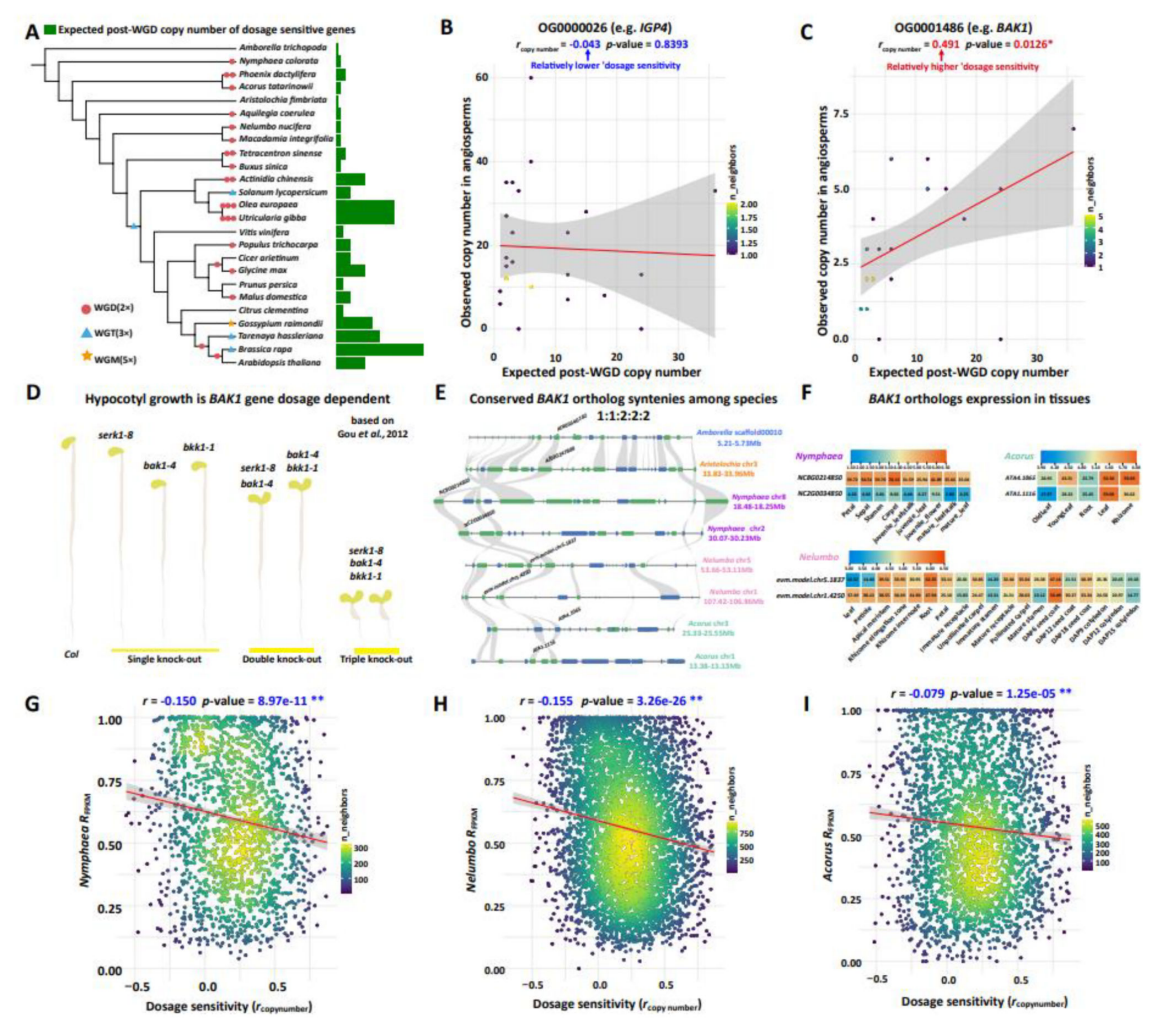
The role of gene dosage in gene expression patterns of homeologs. **A**. Copy number of dosage sensitive genes aligns with number of whole-genome duplication (WGD), whole-genome triplication (WGT), and whole-genome multiplication (WGM) events. Green bars, the relative copy number in relation to *Amborella* (a taxon without WGD since the origin of angiosperm). **B**. Copy number dynamics of ortholog OG0000026 (containing *IGP4*) in angiosperms: lack of significant correlation between observed and expected copy numbers post-WGD events. **C**. Copy number dynamics of ortholog OG0001486 (containing *BAK1*) in angiosperms: presence of significant correlation between observed and expected copy numbers post-WGD events. **D**. Hypocotyl growth in *Arabidopsis* under dark conditions correlates with silencing of more *BAK1* inparalogs (Gou et al., 2012). **E**. Micro-Synteny Patterns of *BAK1*: Consistent 1:1:2:2:2 distribution in *Amborella, Aristolochia, Nymphaea, Nelumbo*, and *Acorus*. **F**. Tissue-specific expression patterns of *BAK1* orthologs in *Nymphaea, Nelumbo*, and *Acorus*. **G-I**. Pearson correlation between relative expression differences (*R*_FPKM_) of duplicate pairs in *Nymphaea* (**G**), *Nelumbo* (**H**), and *Acorus* (**I**) and their dosage sensitivity (*r*_copy number_), as reflected by copy number changes in angiosperms associated with expected post-WGD copy numbers. *p*-value < 0.01 **.

In light of previous studies, suggesting that post-WGD slow-evolving genes are less likely to be lost (Inoue et al., 2015), we hypothesize that duplicate pairs with greater expression balance (less expression divergence) are subject to strong dosage-balance constraints and consequently, orthologs tend to have copy numbers that correlate with the number of experienced WGDs (Tasdighian et al., 2017). For instance, for one copy in *Amborella trichopoda, Vitis vinifera* should have 3 copies and *Brassica rapa* even 36 copies, given their respective number of WGD(s) (Figure 3A, Supplementary Table S6). This observation allows for the measurement of ‘relative dosage sensitivity’ by Pearson correlation analyses. For example, while observed copy numbers in some gene families, like the leucine-rich repeat kinase *IGP4* functioning in plant immunity (OG0000026) (Martín-Dacal et al., 2023), show a lack of significant correlation with the expected post-WGD copy numbers (*r*=-0.043, *p*-value=0.8393) (Figure 3B), other genes such as *BAK1* and its OGs (another group of leucine-rich repeat kinases, OG0001486), essential for various cellular processes including brassinosteroid signaling (Li et al., 2002), displayed a pronounced sensitivity to dosage alterations evidenced by fitting a strong positive linear regression with expected copy numbers post-WGD events (*r*=0.491, *p*-value=0.0126) (Figure 3C). The relatively high dosage sensitivity of *BAK1* is also reflected by a study of *Arabidopsis* where single-, double- and triple-mutants of *BAK1* paralogs (*SERKs*) exhibit more severe reduction of hypocotyl (Gou et al., 2012) and root growth (Ou et al., 2022), emphasizing the critical role of gene dosage in plant developmental processes (Figure 3D). Our analysis of genomic structure and gene expression patterns revealed that the micro-syntenies surrounding *BAK1* are conserved, exhibiting a consistent 1:1:2:2:2 distribution across *Amborella, Aristolochia, Nymphaea, Nelumbo*, and *Acorus* (Figure 3E). Moreover, we noted that *BAK1*'s orthologs show balanced expression in the tissues of *Nelumbo* and *Acorus*, while this balanced expression was not well preserved in *Nymphaea*, likely due to its older WGD age (Zhang et al., 2019) (Figure 3F).

Our correlation analyses reveal a significant negative association between the relative expression differences of duplicate pairs, i.e., average *R*_FPKM_, and their dosage sensitivity in response to WGD events proxied by *r*_copy number_ (observed vs. expected post-WGD copy numbers). This association was consistent across *Nymphaea, Nelumbo*, and *Acorus*, thereby highlighting the universal nature of our findings (Figure 3G-I). Upon quantifying the propensity for gene loss (PGL) of each OG using COUNT software, which performs evolutionary analysis of phylogenetic profiles with parsimony and likelihood (Csűös, 2010), we observed a notable positive correlation between average *R*_FPKM_ and their PGL values (Supplementary Figure S8). This observation further corroborates our hypothesis that the expression balance of homeologs is indicative of gene longevity following WGDs.

**Figure 4:**
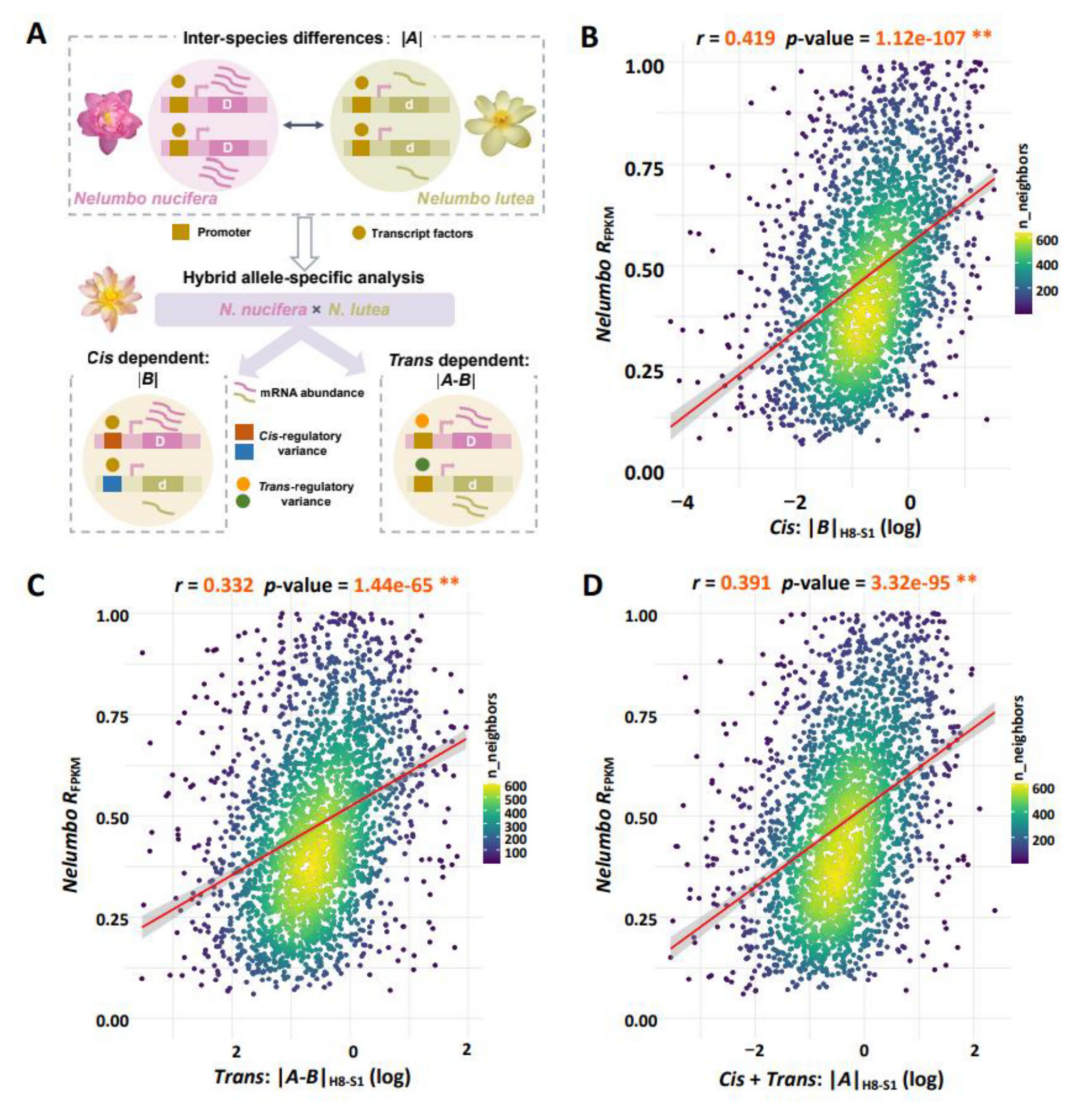
Relationship between *cis*- and *trans*-regulatory variation in homeologs and relative expression differences in *Nelumbo* species. A. Model depicting quantification of *cis*- and *trans*-regulatory changes based on allele-specific expression between *Nelumbo nucifera* and *Nelumbo lutea*. B. Pearson correlation between relative expression difference (*R*_FPKM_) and *cis*-regulatory change magnitude (H8-S1). C. Pearson correlation between *R*_FPKM_ and *trans*-regulatory change magnitude (H8-S1). D. Pearson correlation between *R*_FPKM_ and combined *cis*- and *trans*-regulatory change magnitude (H8-S1). *, *p*-value < 0.05 ; **, *p*-value < 0.01 ; log, log-transformed values of regulatory change magnitude.

Homeologs with greater expression difference show rapid regulatory change between *Nelumbo* species To further our understanding of expression divergence of duplicates resulting from WGD, we considered the role of *cis*- and *trans*-regulatory variations in two *Nelumbo* species, namely *Nelumbo nucifera* and *Nelumbo lutea*, which diverged approximately ~6.5 million years ago. Our hypothesis posits that these regulatory changes are more pronounced in homeologs with high *R*_FPKM_ values given lesser dosage-balance constraints. Through allele-specific expression analysis (ASE) (see Materials and Methods) (Figure 4A) (Gao et al., 2023), we quantified the regulatory changes, uncovering positive correlations between the magnitude of *cis*- and *trans*-regulatory alterations and relative expression differences *R*_FPKM_ for both linear and log-transformed regressions (Pearson correlation test, all *p*-values<0.01), which support this hypothesis (Figures 4B, 4C, 4D, Supplementary Table S7). Thus, *cis-* and *trans*-regulatory mutations uncover a fascinating evolutionary path distinguishing homeologs with balanced expression from those with biased expression. This distinction is further highlighted by a lower frequency of premature stop codon mutations in homeologs exhibiting balanced expression (*R*_FPKM_ ranging from 0 to 0.6), compared to their biased expression counterparts (*R*_FPKM_ exceeding 0.6) within *Nelumbo* populations (Supplementary Figure S9).

Although as mentioned above we conducted correlation tests between *R*_FPKM_ and various factors such as gene features, relative dosage sensitivity, and regulatory divergence using published genome assemblies and annotations, it is unclear to what extent our results are impacted by incomplete genome assembly or annotation. To address this concern, we performed additional correlation tests. Specifically, we simulated scenarios with various degrees of incompleteness by randomly removing 2.5%, 5%, 10%, 20%, and 40% of the *Nelumbo* homeologs from our dataset. The results reveal that these simulated deletions have minimal effect on the correlation coefficient (*r*) (Supplementary Table S8). As the percentage of deletions increases, the p-values show a slight upward trend, but they remain statistically significant even at the highest deletion level of 40% (Supplementary Table S8). Considering these results, along with the high BUSCO scores achieved for the three species studied, we are confident of the adequacy and reliability of our datasets to support our conclusions.

### Divergent evolutionary paths between homeologous copies with low and high expression

**Figure 5:**
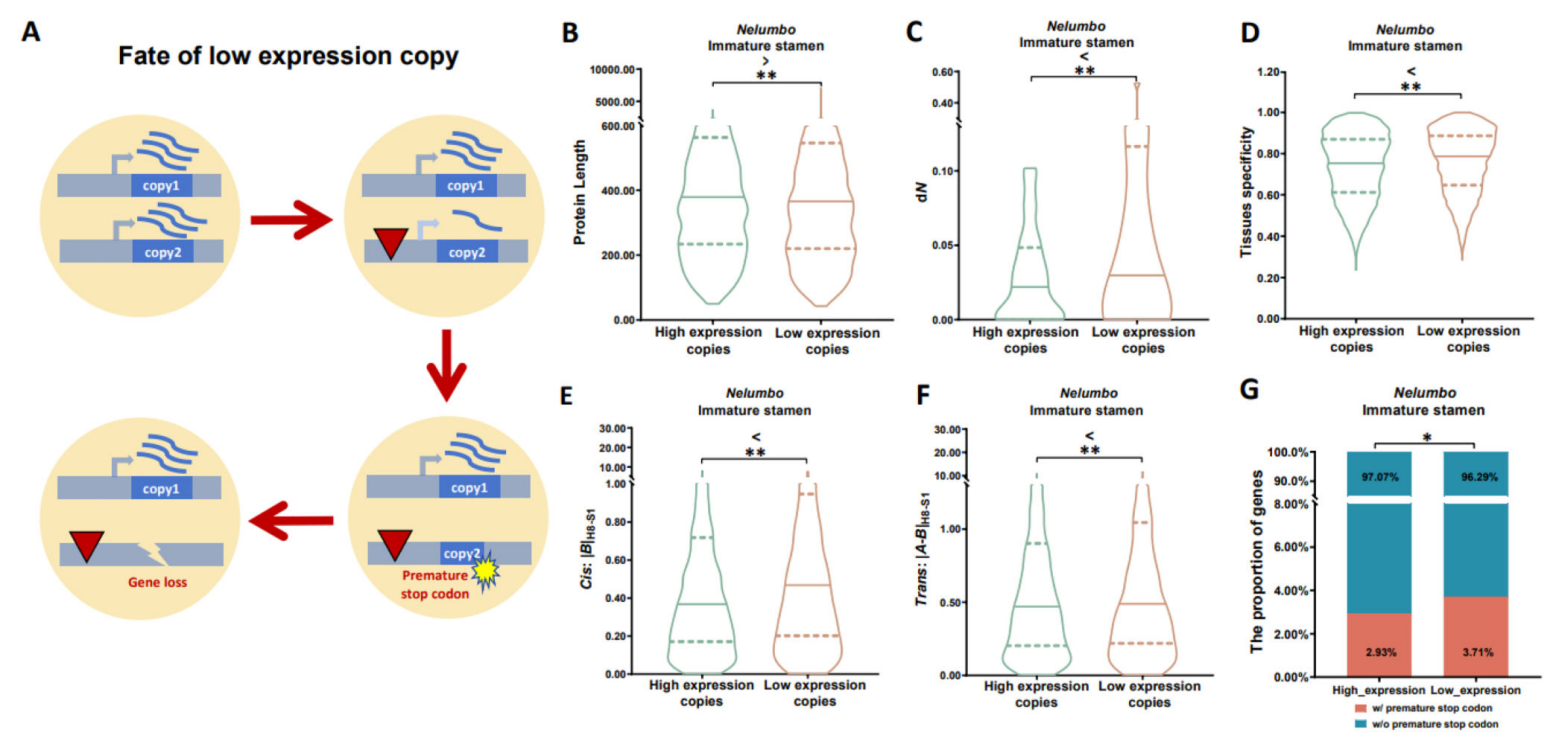
Homeologs with high and low gene expression in immature stamen of *Nelumbo*. **A**. The ‘dead gene walking’ model for the ‘low expression’ copy of homeologs. **B-E**. Comparison between ‘high expression’ and ‘low expression’ copies in protein length (**B**), *ω* (d*N*/d*S* ratio) (**C**), tissue specificity (τ) (**D**), magnitude of *cis*-regulatory mutations (in H8-S1) (**E**), and magnitude of *trans*-regulatory mutations (in H8-S1) (**F**) (paired *t* test, *p*-value <0.01 **). **G**. Comparison between ‘high expression’ and ‘low expression’ copies in proportion of genes with premature stop codon mutations in *Nelumbo* populations (χ^2^ test, *p*-value<0.05 *).

A further analysis was inspired by a study of B_sister_ genes in crucifers (Hoffmeier et al., 2018). B_sister_ genes, belonging to MIKC-type MADS-box genes encoding transcription factors, play a vital role in ovule and seed development (Hoffmeier et al., 2018). Hoffmeier et al. suggested that one ancient gene copy from the core eudicot γ triplication (Jiao et al., 2012; Shi and Chen, 2020), known as a GOA-like gene, has often lost its function and got lost in different plant lineages due to their relatively lower expression, whereas ABS-like genes, derived from the other ancient copy of the B_sister_ genes, are significantly conserved due to their relatively higher expression. These GOA-like genes were therefore referred to as ‘a dead gene walking’, the hypothesis being that varying expression levels in these genes lead to different evolutionary outcomes (Figure 5A). We tested this hypothesis by examining the evolutionary outcomes including rates of non-synonymous (d*N*) and synonymous (d*S*) substitutions, *dN/dS* ratios (*ω*), exon numbers, CDS lengths, protein lengths, Pfam domains, and tissue specificity of expression, in low expression versus high expression homeologous copies in *Nelumbo* (Figure 5B-D, Supplementary Figures S9, Supplementary Table S9). Significant differences observed (*p*-value<0.01), as per the paired *t* tests, affirmed our hypothesis of higher expression copies tending to be more conserved in terms of sequence substitutions and gene structure, showing broader expression breadth. The exceptions observed in the number of exons and Pfam domains could be attributed to the intrinsic structural and functional constraints of these genomic elements (Supplementary Figures S9, Supplementary Table S9), which may be less susceptible to evolutionary changes driven by differential expression levels. The trend mirrored in *Nymphaea* and *Acorus* suggests a broader applicability of this evolutionary pattern (Supplementary Figures S10-S11, Supplementary Table S9). Thus, homeologous copies with relatively higher expression levels are subject to stronger selective constraints, thus retaining essential functions, resisting mutations and nonfunctionalization.

Building on this hypothesis, we also considered *cis*- and *trans*-regulatory changes of homeologs between the closely related species *Nelumbo nucifera* and *N. lutea*. Consistent with the B_sister_ gene narrative, our results revealed significant differences in the magnitude of *cis*- and *trans*- regulatory changes between higher and lower expression homeologs (Figure 5E-F, Supplementary Figures S9, Supplementary Table S10). The lower magnitudes of *cis*- (measured by |*B*|) and *trans*- regulatory mutations (measured by |*A*-*B*|) (see Materials and Methods) in higher expression copies suggest that conservation and higher gene expression levels are intertwined. This is also particularly evident in the reduced incidence of premature stop codon mutations in these highly expressed copies, suggesting an ongoing selective process preventing gene copies with higher expression from nonfunctionalization (χ^2^ test, *p*-value<0.01) (Figure 5G, Supplementary Figures S12).

## Discussion

We considered three angiosperms, each having undergone a single, independent WGD, since the origin of angiosperms, namely *Nymphaea* (a WGD of 117–98 MYA) (Zhang et al., 2019), *Nelumbo* (a WGD about 65 MYA) (Ming et al., 2013; Shi et al., 2020), and *Acorus* (a WGD about 41.7 MYA) (Shi et al., 2022; Ma et al., 2023). Despite one or two older WGDs preceding the origin of angiosperms (Jiao et al., 2011; Ruprecht et al., 2017), our intra-specific synteny search using MCScanX revealed only 127 homeologous pairs in *Aristolochia*, with none in *Amborella*, two species without a lineage-specific WGD (Amborella Genome, 2013; Qin et al., 2021). This also implies that the number of homeologs resulting from one or two older WGDs (be it in the ancestral angiosperm and/or ancestral seed plant lineage) will be very limited in *Nymphaea, Nelumbo*, and *Acorus*, and not affecting the analysis described here. Differences in the number of homeologs identified in each of the three species used can be attributed to several factors including the age differences between the three WGDs, as well as the numbers of ancestral genes present before each WGD, and lineage-specific gene loss rates. Hence, the uniformity in the date of origin of the majority of homeologs – because identified through synteny analysis suggesting large-scale duplication - enables us to confidently attribute any observed expression divergence between homeologous copies to the varying strengths of dosage-balance constraint, the focal point of our study. This approach precludes confounding factors associated with species that have undergone multiple WGDs, such as Arabidopsis, or smaller-scale gene duplicates like tandem duplicates, where the origins are more dispersed in time, rendering it difficult to discern if expression divergence is driven by dosage-balance constraint, or simply the passage of time.

Careful analysis showed that gene expression evolution of homeologs in these *Nymphaea, Nelumbo*, and *Acorus* — whether balanced or unbalanced between copies— is not random. Instead, it seems determined by dosage balance constraints. Expression divergence in duplicate genes is often observed between gene copies following gene duplication or WGD events. One archetype of this divergence is the scenario where one copy exhibits significantly higher expression than its counterpart. Such biased expression level between copies is often linked to the ‘subgenome dominance’ phenomenon and can be attributed to various factors, including mutations in regulatory regions, methylations, chromatin accessibility, and changes in transcription factor affinities, acting different in different subgenomes (Li et al., 2005; Cheng et al., 2016; Zhao et al., 2017; Bird et al., 2021; Garcia-Lozano et al., 2021; Li et al., 2022). As our study reveals, it becomes increasingly evident that the post-WGD evolutionary trajectory of gene expression is not uniformly characterized by divergence.

Remarkably, a subset of gene pairs maintains similar expression levels over extensive evolutionary timescales. This finding complements the widely accepted hypothesis that redundant gene copies are preserved primarily through subfunctionalization or neofunctionalization. In quantifying expression bias between copies using *R*_FPKM_, we observe a convergent pattern among orthologs across different species. This pattern suggests a selective pressure influencing expression evolution. It seems that convergence in expression levels may be driven by factors (gene features) such as protein lengths, number of protein domains, and number of protein-protein interactions. These gene features might indirectly relate to dosage-balance constraints. A protein domain is a distinct, conserved region within a protein that can independently fold and function, with multi-domain proteins often being longer and potentially more prone to diverse protein-protein interactions (Zhang and Yang, 2015). Additionally, lower lethal-phenotype scores in *Arabidopsis* orthologs for homeologs with balanced expression further implies strong selection against expression variation. Indeed, homeologs with balanced expression levels are likely subjected to more stringent stoichiometric constraints. As a result, these homeologs with highly balanced expression levels are likely more susceptible to purifying selection. The lethal-phenotype score serves as an alternative metric to assess the constraint imposed on genes, complementing ‘protein structure’-related measures. In a study focused on *Arabidopsis*, each gene was assigned a lethal-phenotype score ranging from 0 to 1 (Lloyd et al., 2015). In this scoring system, higher values correspond to a greater probability of exhibiting lethal phenotypes when the gene is disrupted. This provides a quantifiable way to understand a gene's essentiality. Lower expression tissue specificity for homeologs with balanced expression is also supported by the association between broader gene expression and higher essentiality that has been observed earlier (Liao et al., 2006). Furthermore, these genes are characterized by lower rates of non-synonymous (amino acid) sequence substitutions and a broader expression range across tissues, also indicating strong purifying selection (Kondrashov et al., 2002; Cardoso-Moreira et al., 2016). Our observed increase in the number of exons for genes that show balanced expression points to a more complex gene structure, potentially enabling diverse functions (Zhang, 2003; Van de Peer et al., 2009). In eukaryotes, there is a positive correlation between the number of exons and protein domains (Liu and Grigoriev, 2004), which means that moa higher number of exons might lead to a higher number of protein-protein interactions (PPIs), and therefore we also incorporated the number of exons as an extra variable. This overall gene complexity may result in increased functional constraints and dosage balance, a hypothesis supported by previous studies (Carels and Bernardi, 2000; Bowers et al., 2022). We realized that, in our study of *R*_FPKM_, there are differences in dataset extensiveness among species, particularly for *Nelumbo* because its genome was published already in 2013, whereas the genomes of *Nymphaea* and *Acorus* were published in 2019 and 2022, respectively. However, congruent correlation tests and other statistical analyses of different tissues and species, ensure that our conclusions are consistent despite differences in dataset size. Overall, our research thereby adds a significant layer to our understanding of gene expression dynamics of duplicate genes in the context of evolutionary genomics (Pal et al., 2001; Conant and Wagner, 2003; Jordan et al., 2005; Holland et al., 2017). We acknowledge that some features, like the average number of Pfam domains and the Lethal_phenotype score of Arabidopsis OGs, show weaker correlations with *R*_FPKM_. However, we believe that this is reasonable given that sequence divergence and tissue expression specificity exhibit a much broader range of variance, allowing for precise characterization of genes. Conversely, the Lethal_phenotype score and the number of Pfam domains are estimated more crudely, based on homologous gene transfer and annotations of known domains, and exhibit a limited range of variance. The lethal-phenotype score and the number of Pfam domains, thus, serve as auxiliary parameters, providing additional support for reflecting the levels of dosage balance constraint on genes. Such variations in correlations are also commonly observed in studies like GWAS, where there are leading loci and ‘suboptimal’ loci differentially associated with target traits. Overall, we believe that all gene features analyzed point to the significant role of dosage balance constraint in shaping expression divergence between homeologous copies.

Several studies demonstrated that dosage-sensitive genes are slow in regulatory change after WGDs. For example, a study on diploid and polyploid *Glycine* species suggests that duplicates from different rounds of WGDs and annotated with ‘metabolic pathways’ and Gene Ontologies that are putative dosage sensitive exhibit reduced expression variance across the species compared to those putative dosage insensitive genes. This indicates a tendency towards stabilized, less variable gene expression in response to WGDs for dosage sensitive genes (Coate et al., 2016). Another study revealed that following ploidy changes in *Arabidopsis*, genes sensitive to dosage balance showed more coordinated transcriptional responses (similar expression alterations) than dosage-insensitive genes and less variable of expression level among accessions, indicating that gene expression regulation and duplicate gene retention are influenced by selection for dosage balance rather than simple gene dosage increase (Song et al., 2020). Thus, our hypothesis centers upon the premise that dosage-sensitive genes, characterized by their slow expression change during species divergence as exemplified in *Glycine* and *Arabidopsis*, can maintain balanced expression levels between homeologs even after extensive periods of paleopolyploidy, spanning tens of millions of years. Consequently, we posited and tested that the persistence of balanced gene expression in homeologs serves as an indicator of high dosage sensitivity and gene longevity, a key aspect we have further examined in our current study. To conclude, the retention of duplicate genes with balanced expression serves as a mechanism to buffer against disruption of functional integrity of stoichiometry of interacting proteins.

Dosage balance constraints affect more than just gene expression (Birchler and Veitia, 2012). In plants, vertebrates, yeast, and other organisms, a common pattern emerges where the fate (retention or deletion) of duplicated genes post WGD is influenced by factors such as dosage sensitivity (Birchler and Veitia, 2021). In vertebrates, the selective constraints on coding sequences of nervous system genes significantly influence duplicate gene retention, particularly after WGD, due to the need for purifying selection against protein misfolding or misinteracting in nonrenewable neural tissues (Roux et al., 2017). In yeast, whole-genome duplicate gene retention is shaped by complex genetic interactions, revealing that duplicated genes often have entangled functions and their retention is influenced by factors like gene expression, protein interactions, and evolutionary age (Kuzmin et al., 2020). In angiosperms, gene duplicability across 37 surveyed species showed to be remarkably similar, even in the context of independent WGDs, indicating a potential sensitivity of these genes to dosage balance (Li et al., 2016). The variation in WGD episodes among different angiosperm lineages (Van de Peer et al., 2017) implies that for genes sensitive to dosage changes, the number of copies in related species should align with those produced by their historical WGDs or WGMs (whole genome multiplications). Indeed, reciprocally retained genes after WGDs or WGMs in angiosperm lineages exhibit dosage balance sensitivity, based on functional annotations, characterized by stronger sequence divergence constraints and lower rates of functional and expression divergence compared to other putative dosage insensitive genes (Tasdighian et al., 2017). In alignment with these studies, we found that homeologs with balanced expression typically not only show a copy number of orthologs (genes in different species that evolved from a common ancestral gene) consistent with the expected numbers post-WGD but also a lower propensity of gene loss during angiosperm radiation. For example, *BAK1* and its related *SERK* homologs, essential leucine-rich kinases in plants, interact with *BRI1* for brassinosteroid signaling essential for plant growth, G proteins for sugar signaling, and *BTL2* in immune responses (Li et al., 2002; Liu et al., 2020). The correct gene dosage of these kinases is critical, as altering their numbers significantly impacts plant growth (Gou et al., 2012). Our study also shows the dosage sensitivity of *SERKs* in angiosperms, underscoring their importance in plant biology. Thus, our research indicates that maintaining expression balance of homeologs is indicative of gene essentiality and persistence following any independent WGD events in plants. However, interestingly, we found that *SERK*s are missing in some species like the carnivorous *Utricularia gibba* (floating bladderwort) and the desert-dwelling *Phoenix dactylifera* (date palm). We searched for orthologs of *SERK*s in the Plant Plaza 5.0 database (Van Bel et al., 2022), and found that SERKs is also absent in *Elaeis guineensis* (oil palm).

More intriguingly, in our study on the evolution of *Nelumbo* species within the last ~6.5 million years (see http://www.timetree.org), we have uncovered a distinct evolutionary trajectory for homeologs characterized by balanced versus biased expression. Our observations reveal that homeologs with balanced expression between *N. nucifera* and *N*. *lutea* exhibit fewer *cis*- and *trans*-regulatory mutations. Additionally, homeologs showing balanced expression demonstrate a reduced incidence of pre-mature stop codon mutations within populations, compared to their biased expression counterparts. This finding highlights the significant impact of expression balance on the evolutionary dynamics of gene variants at the population level in *Nelumbo* species, suggesting ongoing escalation of duplicate divergence. Meanwhile, our observations that homeologs of balanced expression with higher frequency of pre-mature stop codon mutations suggest that balanced expression in homeologs might act as a stabilizing factor, mitigating the accumulation of deleterious mutations.

For duplicate genes, the lesser expressed copy often culminates in the nonfunctionalization due to relaxed selective pressures, leading to the accumulation of deleterious mutations and eventual loss of function (Innan and Kondrashov, 2010). In the current study, we discovered that gene copies with higher expression levels exhibit broader tissue expression, longer protein structures, and notably, a lower d*N*/d*S* ratio, indicating stronger purifying selection. This unique selection pressure is further corroborated by micro-evolutionary patterns observed in *Nelumbo* species divergence. Specifically, in the divergence between *N. lutea* and *N. nucifera*, we noted a reduced magnitude of both *cis*- and *trans*-regulatory mutations and a lower incidence of premature stop codon mutations in the higher expression gene copies. This suggests an ongoing process of gene loss in the copies with lower expression. Such findings not only confirm our initial hypothesis but also provide a nuanced understanding of how expression levels dictate the occurrence of regulatory variation and the evolutionary fate of gene duplicates in plant genomes, mirroring the conservation and functional significance seen in the case study of B_sister_ gene homeologs in crucifers (Hoffmeier et al., 2018). A recent study on *Drosophila* and human genomes suggests that ‘complete’ duplicate genes, which maintain all exons and introns, are subject to dosage constraints due to protein stoichiometry, thereby reinforcing the correlation between a greater protein length in highly expressed copies in our study (Zhang et al., 2022a). Also, our observation of distinctiveness between copies is in accordance with the general assumption that slower evolving genes are more conserved and often exhibit higher expression levels and greater functional importance (Pal et al., 2001; Conant and Wagner, 2003; Jordan et al., 2005; Holland et al., 2017). Notably, a typical example demonstrated that while *ABS*-like genes, a clade of B_sister_ genes, are highly conserved in crucifers and maintain their ancestral function in ovule and seed development, their closest paralogs from core eudicot γ triplication, the *GOA*-like genes, are experiencing convergent down-regulation or gene death in *Brassicaceae* (Hoffmeier et al., 2018). Thus, the trend towards nonfunctionalization of the lesser expressed gene copies suggests a predominant evolutionary strategy where plants retain only the necessary gene functions for survival, shedding redundant copies. This process significantly influences the genomic architecture and functional repertoire of plant species, as observed in multiple recent genomic studies (Carretero-Paulet and Van de Peer, 2020; Zhong et al., 2022; Gout et al., 2023). It is important to acknowledge that while there is a noticeable trend of unequal fates for copies with lower expression, their eventual loss is not inevitable. In some cases, these copies may persist for extended periods if they acquire new functions or regulatory mechanisms (neofunctionalization), or partitioning ancestral functions or expression (subfunctionalization) as exemplified by investigations in *Arabidopsis* (Panchy et al., 2019; Coate et al., 2020; Jonas et al., 2022). Overall, such insights are indispensable for unraveling the complex evolutionary processes shaping plant genomes, furthering our understanding of plant biodiversity and adaptation strategies.

## Materials and methods

### Detection of homeologs

To elucidate the expression level difference and dosage balance constraints between duplicate gene pairs originating from whole genome duplications (WGDs), i.e., homeologs, we curated a comprehensive dataset of WGD-derived duplicate gene pairs (Tiley et al., 2016). This dataset encompasses genes from *Nymphaea colorata* (Zhang et al., 2019), *Nelumbo nucifera* (Ming et al., 2013; Shi et al., 2020), and *Acorus tatarinowii* (Shi and Chen, 2020; Shi et al., 2022). The identification of these gene pairs was conducted using MCSCanX with parameter settings of ‘-s 6’ (minimum of six anchor genes per block) (Tang et al., 2008; Wang et al., 2012). Furthermore, we utilized the 'detect_collinear_tandem_arrays' function in MCSCanX to identify anchor gene pairs associated with tandem arrays. In cases where two or more anchor gene pairs are associated with the same tandem array, we only kept the anchor gene pair with the lowest e-value. To advance our understanding of the post-WGD evolutionary trajectory of these homeologs, we established an approach where we compared, for each species, the duplicate pairs with an outgroup. This entailed the construction of syntenic blocks between *Nelumbo nucifera* and *Macadamia integrifolia* (Proteales), *Acorus tatarinowii* and *Phoenix dactylifera* (monocots), and *Nymphaea colorata* and *Aristolochia fimbriata* (ANITA-grade). The identification of syntenic orthologs within these blocks was executed utilizing MCScanX (Tang et al., 2008; Wang et al., 2012). In instances where duplicates lacked a syntenic ortholog in the outgroup species, ortholog identification was achieved via a comprehensive analysis of potential protein sequences using OrthoFinder (v2.3.3) with default settings (Emms and Kelly, 2019). Following the integration of data from both OrthoFinder and MCScan, we successfully compiled a dataset of high-confidence gene triplets, each consisting of duplicate gene pairs and their corresponding outgroup orthologs.

Quantification and tests of relative expression difference of duplicate pairs from a WGD

We established gene expression profiles for multiple tissues in *Nymphaea* (Zhang et al., 2019), *Nelumbo* (Li et al., 2021a; Li et al., 2021b; Zhang et al., 2022b; Gao et al., 2023), and *Acorus* (Shi et al., 2022; Ma et al., 2023), utilizing RNA-seq datasets in the cited manuscripts (Supplementary Table S1). For each species, we processed the RNA-seq reads by mapping them to their respective reference genomes using Hisat2 (v2.1.0) (Pertea et al., 2016). The resulting SAM files were then sorted, converted to BAM format, indexed, and underwent PCR duplicate marking. This was achieved using Samtools (v0.1.19) and Picard (version 2.0.1). Subsequent gene annotation was aligned with existing gene annotations. We quantified gene expression levels, denoted as FPKMs, employing StringTie (v1.3.5) with default parameter settings (Pertea et al., 2016). For each pair of homeologs, we determined the relative expression differences between the two copies. This was accomplished by calculating the absolute normalized difference in expression for each tissue type, a metric we refer to as *R*_FPKM_. This approach follows the principles outlined in previous studies (Conant and Wagner, 2003; Cusack and Wolfe, 2006) :

$$R_{FPKM}=\frac{|{\text{Expression}}_{\text{copy1}}-{\text{Expression}}_{\text{copy2}}|}{{\text{Expression}}_{\text{copy1}}+{\text{Expression}}_{\text{copy2}}}$$

To assess the consistency of *R*_FPKM_ values of duplicate genes across different tissues, we conducted Pearson correlation tests (Supplementary Table S1). These tests compared tissue-specific *R*_FPKM_ values within the same species using *R* (v3.5.1) (https://www.r-project.org/). For each duplicate pair, we averaged the *R*_FPKM_ values across all tissues for subsequent analysis. Additionally, we explored the relationship between the average *R*_FPKM_ of homeologs and various gene characteristics via regression analyses using *R* (v3.5.1). We compared linear and log-transformed regressions based on their AIC values in *R* (v3.5.1) to determine the best fit for gene characteristics that may not exhibit a linear relationship with *R*_FPKM_. We chose log regression when it yielded a lower AIC. These characteristics include tissue specificity (τ) of gene expression, protein and CDS lengths, number of exons, number of Pfam domains, number of protein-protein interactions (PPIs) (Yilmaz et al., 2022), and lethal-phenotype scores (Lloyd et al., 2015) derived from *Arabidopsis* orthologs. We also considered nonsynonymous divergence (d*N*), synonymous divergence (d*S*), and the d*N*/d*S* ratio (*ω*). The measurements for protein and CDS lengths, as well as number of exons, were extracted directly from each species' genome annotation file.

The Pfam domain count per gene was based on annotations via emapper-2.1.12 (Cantalapiedra et al., 2021). The τ index, a benchmark of gene expression tissue-specificity metrics, for each gene was calculated using log-transformed FPKM values from different tissues) (Kryuchkova-Mostacci and Robinson-Rechavi, 2016; Shi et al., 2020; Gao et al., 2023). The computations of d*N*, d*S*, and *ω* were performed using the codeml program within the PAML4 package, following a triplet tree topology of ‘((copy1,copy2),outgroup)’ (Yang, 2007). To assess the possible effects of incomplete genome assembly or annotation on our regression analyses, we performed extra correlation tests using *Nelumbo*. These tests involved simulating different levels of incompleteness by randomly removing 2.5%, 5%, 10%, 20%, and 40% of *Nelumbo*'s total homeologs from our dataset through ‘sample()’ function in *R* (v3.5.1). To investigate the variation of average *R*_FPKM_ values among different Gene Ontology (GO) slim categories, we categorized different homeologs into TAIR GO slim categories (TAIR_GO_slim_categories.txt from https://www.arabidopsis.org). This categorization was based on the GO annotations of genes obtained via emapper-2.1.12 (Cantalapiedra et al., 2021). We then visualized the distribution of average *R*_FPKM_ values across these GO slim categories using violin plots, created with Graphpad Prism 9.0.

### Assessing dosage sensitivity by copy number change

Orthologous groups from 25 representative angiosperm species, including *Nymphaea, Nelumbo*, and *Acorus*, were identified using OrthoFinder (v2.3.3) with default settings (Emms and Kelly, 2019) (see Supplementary Table S3). The expected gene copy number in these lineages, accounting for their respective historical WGDs or WGMs, was determined based on existing literature (Supplementary Table S3). We quantified the relative dosage-sensitivity of an orthologous group (OG), referred to as *r*_copy number_, by determining the (Pearson) correlation coefficient. This coefficient was calculated between the observed and expected copy numbers of genes following whole-genome duplications (WGDs) or whole-genome multiplications (WGMs) (see Supplementary Table S3). To further validate the relative dosage sensitivity of different OGs, we calculated the Krylov-Wolf-Rogozin-Koonin ‘propensity for gene loss’ (PGL) for each OG by using the COUNT software (Csűös, 2010). Additionally, we conducted an analysis to explore the relationship between the average *R*_FPKM_ of homeologs and their PGL. This was achieved by calculating the Pearson correlation between these two parameters. The calculations and analyses were performed using *R* (v3.5.1).

### Regulatory changes and premature stop codon mutations associated with different homeologs

We sought to determine if there is a positive correlation between the *R*_FPKM_ of homeologs and the magnitude of *cis*- and *trans*-regulatory variations between *Nelumbo nucifera and N. lutea*. To do this, we compared three key values—absolute *A, B*, and |*A*-*B*|. Here, '*A*' denotes the parental expression difference, '*B*' represents the *cis*-regulatory difference, and '|*A*-*B*|' indicates the *trans*-regulatory difference. These definitions and values were based on our previous research focused on the divergence in petal color between *N. nucifera* and *N. lutea* across four developmental stages (Gao et al., 2023). Furthermore, we investigated whether different homeologs with varying *R*_FPKM_ values exhibit distinct frequencies of premature stop codon mutations. This was carried out by analyzing SNP annotations in *Nelumbo* populations. These annotations were obtained using SnpEff (Version 4.3) and were extracted from our prior studies (Huang et al., 2018; Li et al., 2021b). Further, for each species, we categorized homeologs within each tissue into two groups based on their expression levels: low expression and high expression copies. We then compared the evolutionary trajectories between these two groups through either paired *t* test or χ^2^ test via Graphpad Prism 9.0. This comparison encompassed a range of gene characteristics, including synonymous (d*N*) and non-synonymous (d*S*) substitutions, d*N*/d*S* ratios (*ω*), number of exons, CDS lengths, protein lengths, and Pfam domains. Additionally, we examined the magnitude of cis- and trans-regulatory variations, as well as the frequency of premature stop codon mutations among these groups.

## Supplementary Material

Supplementary Figures

Supplementary Tables

## Figures and Tables

**Table 1:** Pearson correlations between relative expression difference of homeologs (average *R*_FPKM_) and their gene features.

	Gene features	r	*p-*value	confidence interval (lower)	confidence interval (upper)	t	df	AIC
*Nymphaea* average *R*_FPKM_	Average d*N* (log)	0.3161	0	0.2767	0.3544	15.138	2064	457.58
	Average d*S* (log)	0.2115	0	0.1700	0.2523	9.839	2066	581.10
	Average *ω*	0.0597	0.0065	0.0166	0.1026	2.720	2064	667.71
	Average exon number (log)	-0.1000	2.93E-06	-0.1415	-0.0582	-4.6874	2171	711.65
	Average CDS length (log)	-0.0934	3.96E-05	-0.1375	-0.0490	-4.1190	1924	478.25
	Average protein length (log)	-0.1022	1.80E-06	-0.1436	-0.0604	-4.7873	2171	710.72
	Average Pfam	-0.0406	0.0854	-0.0868	0.0056	-1.7208	1789	551.32
	Average tissues specificity	0.1454	9.52E-12	0.1040	0.1863	6.8508	2171	687.06
	Average PPI (log)	-0.0990	0.00028	-0.1519	-0.0456	-3.6348	1333	338.05
	Lethal_phenotype score of *Arabidopsis* OGs (log)	-0.0527	0.0303	-0.1002	-0.0050	-2.1672	1682	350.21
*Nelumbo* average *R*_FPKM_	Average d*N* (log)	0.4125	0	0.3891	0.4353	31.848	4946	-661.21
	Average d*S* (log)	0.2768	0	0.2509	0.3023	20.261	4946	-132.76
	Average *ω* (log)	0.1920	0	0.1650	0.2186	13.759	4946	75.93
	Average exon number (log)	-0.1635	1.90E-31	-0.1904	-0.1365	-11.743	5015	159.23
	Average CDS length	-0.1482	4.89E-26	-0.1751	-0.1210	-10.612	5015	183.91
	Average protein length	-0.1482	4.89E-26	-0.1751	-0.1210	-10.612	5015	183.91
	Average Pfam	-0.0633	3.38E-05	-0.0931	-0.0334	-4.149	4278	252.99
	Average tissues specificity	0.5204	0	0.4999	0.5403	43.159	5015	-1289.3
	Average PPI (log)	-0.1544	2.86E-19	-0.1874	-0.1211	-9.0292	3334	211.42
	Lethal_phenotype score of *Arabidopsis* OGs	-0.0879	1.59E-09	-0.1162	-0.0594	-6.0467	4695	188.14
*Acorus* average *R*_FPKM_	Average d*N* (log)	0.4453	0	0.4177	0.4722	28.654	3318	44.465
	Average d*S* (log)	0.4447	0	0.4170	0.4716	28.602	3318	46.855
	Average *ω* (log)	-0.0984	1.30E-08	-0.1320	-0.0646	-5.7001	3318	746.21
	Average exon number (log)	-0.1851	9.13E-28	-0.2173	-0.1526	-11.017	3418	761.01
	Average CDS length	-0.1583	1.21E-20	-0.1908	-0.1254	-9.3754	3418	793.53
	Average protein length	-0.1583	1.21E-20	-0.1908	-0.1254	-9.3754	3418	793.53
	Average Pfam	-0.0633	3.38E-05	-0.0931	-0.0334	-4.1499	4278	252.99
	Average tissues specificity	0.2870	0	0.2560	0.3175	17.520	3418	586.23
	Average PPI (log)	-0.1418	6E-12	-0.1813	-0.1017	-6.909	2327	546.63
	Lethal_phenotype score of *Arabidopsis* OGs	-0.1388	5.21E-15	-0.1729	-0.1043	-7.8603	3144	588.62

*r*: correlation coefficient; *df*, degree of freedom; AIC, Akaike Information Criterion.

## Data Availability

Data about *Nelumbo, Acorus* and *Nymphaea* analyzed in this study are public and cited in the manuscript.
