# Supplementary material for: Balanced gene expression of homeologs suggests strong dosage sensitivity and gene longevity after paleopolyploidies in angiosperms
Source: Plant Cell. Author manuscript; Available in PMC 2025 Apr 3. (PMC7616505; doi:10.1093/plcell/koae227)
Supplement: Supplementary Figures [file EMS198101-supplement-Supplementary_Figures.pdf]

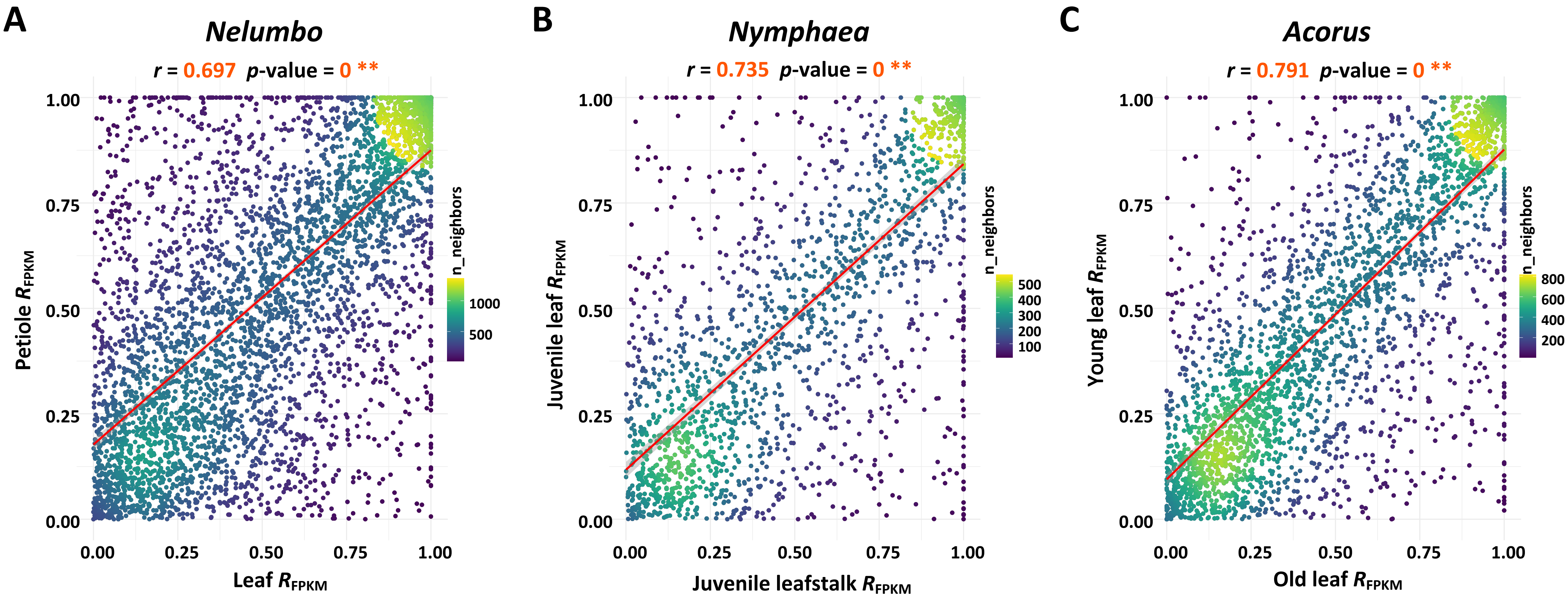

Supplementary Figure S1. Pearson correlations of relative expression differences (R_{FPKM}) of WGD duplicates between different tissues. **A.** Between leaf and petiole in *Nelumbo*. **B.** Between juvenile leaf and juvenile leafstalk in *Nymphaea*. **C.** Between young leaf and old leaf in *Acorus*. WGD, whole genome duplication; FPKM, fragments per kilobase of exon model per million mapped fragments; grey shading band, the 95% (default) confidence level interval for predictions from a linear model; red font, positive correlation; **, *p*-value < 0.01.

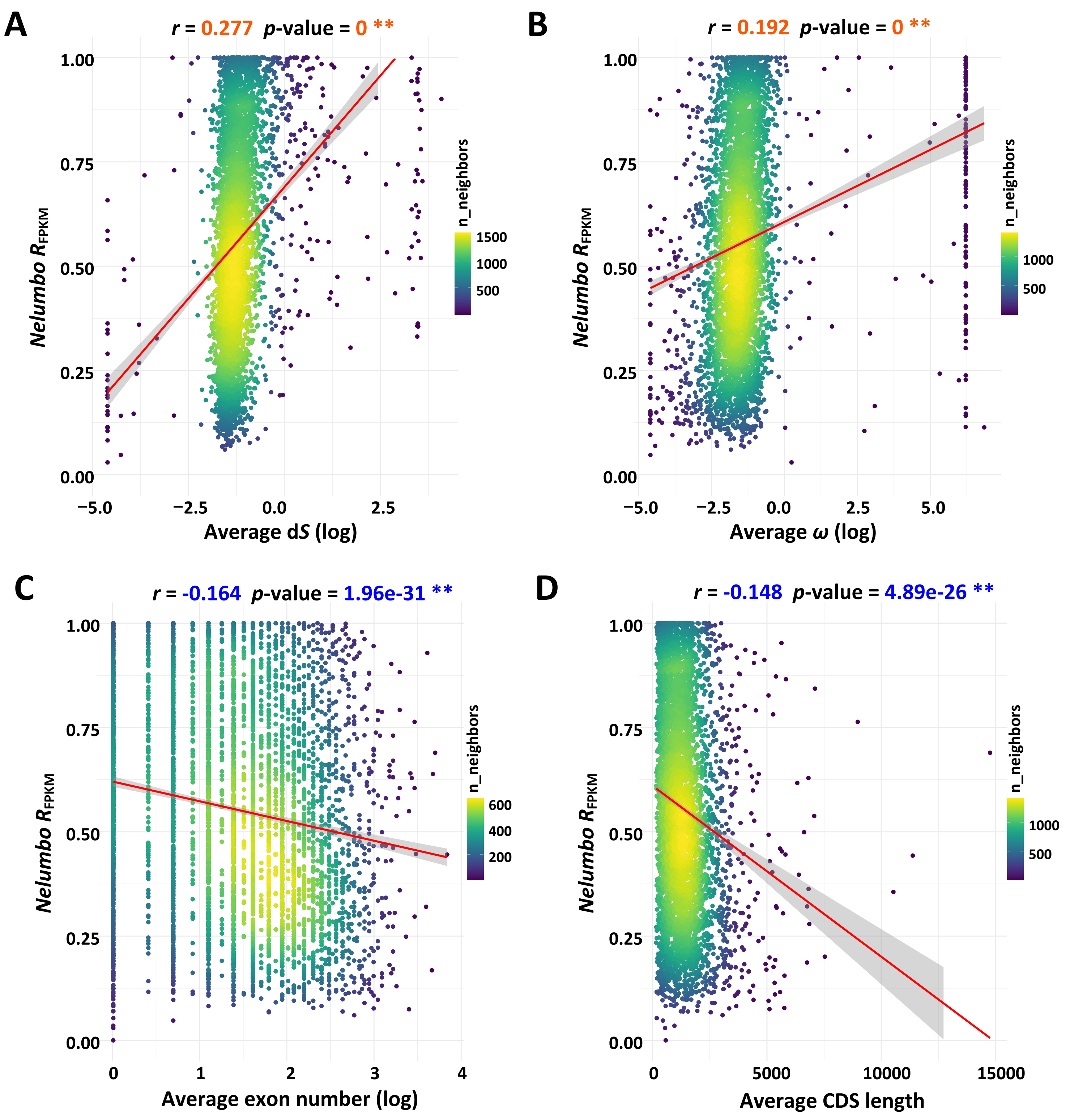

Supplementary Figure S2. Pearson correlations between average relative expression differences (R_{FPKM}) of WGD duplicates and gene features in *Nelumbo*. A-B. Average R_{FPKM} of WGD duplicates are significantly positively correlated with average synonymous substitutions per site (d*S*) after WGD **(A)** and average *ω* (d*N*/d*S* ratio) after WGD **(B)**. **C-D.** Average R_{FPKM} of WGD duplicates are significantly negatively correlated with average exon number **(C)** and average coding sequence (CDS) length **(D)**. WGD, whole genome duplication; FPKM, fragments per kilobase of exon model per million mapped fragments; *r*, correlation coefficient of Pearson correlation test; log, log-transformed values of gene features in x-axis; grey shading band, the 95% (default) confidence level interval for predictions from a linear model; red font, positive correlation; blue font, negative correlation; **, *p*-value < 0.01.

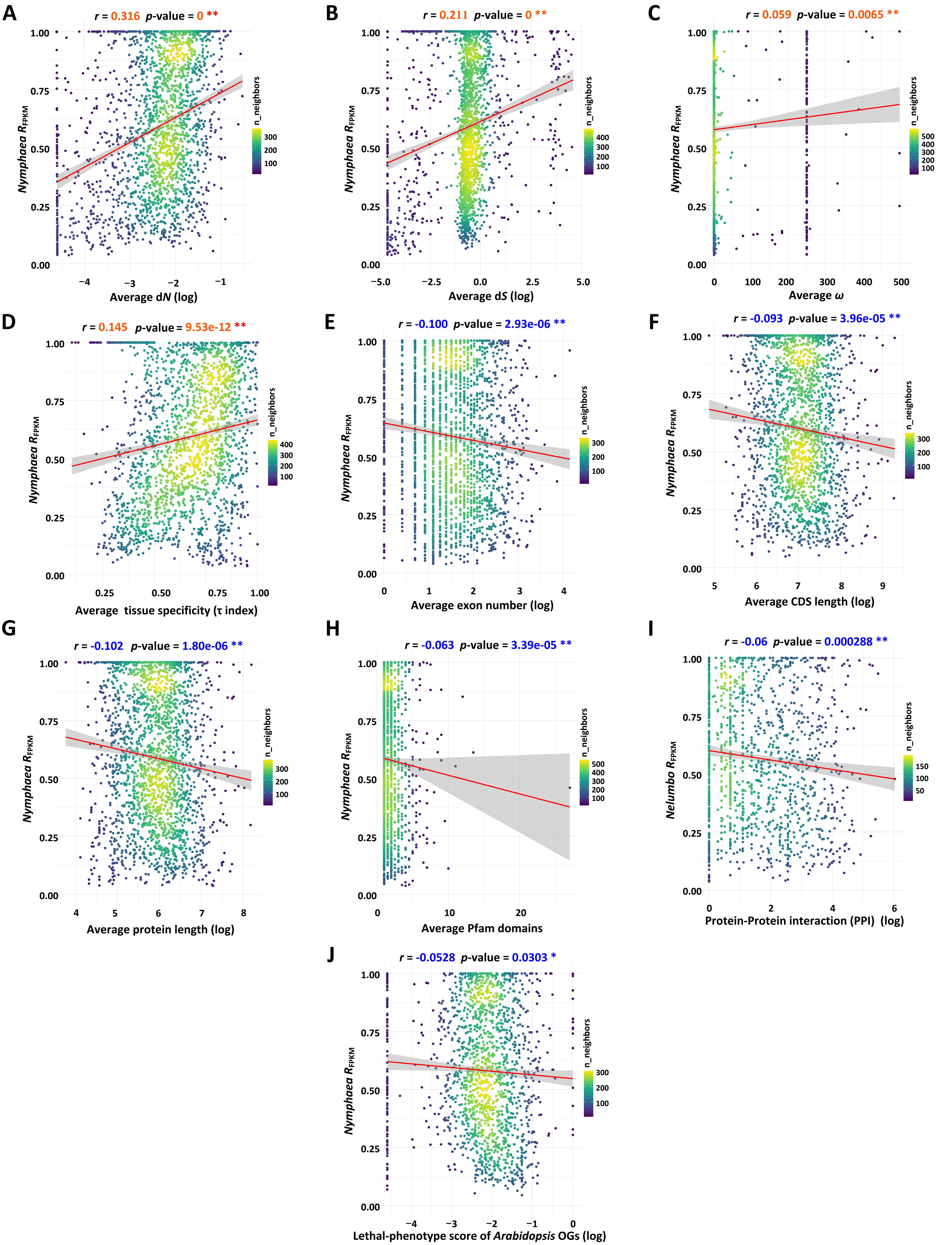

Supplementary Figure S3. Pearson correlations between average relative expression differences (R_{FPKM}) of WGD duplicates and gene features in *Nymphaea*. A-D. Average R_{FPKM} of WGD duplicates are significantly positively correlated with average non-synonymous substitutions per site (d*N*) after WGD **(A)**, average synonymous substitutions per site (d*S*) after WGD **(B)**, average *ω* (d*N*/d*S* ratio) after WGD **(C)**, tissue specificity (τ) of gene expression **(D)**. **E-J.** Average R_{FPKM} of WGD duplicates are significantly negatively correlated with average exon number **(E)**, average coding sequence (CDS) length **(F)**, average protein length **(G)**, No. of Pfam domains genes **(H)**, No. of protein-protein interactions (PPIs) of putative orthologs in *Arabidopsis* **(I)**, lethal-phenotype score of *Arabidopsis* putative ortholog groups **(J)**. WGD, whole genome duplication; FPKM, fragments per kilobase of exon model per million mapped fragments; *r*, correlation coefficient of Pearson correlation test; log, log-transformed values of gene features in x-axis; grey shading band, the 95% (default) confidence level interval for predictions from a linear model; red font, positive correlation; blue font, negative correlation; *, *p*-value < 0.05; **, *p*-value < 0.01.

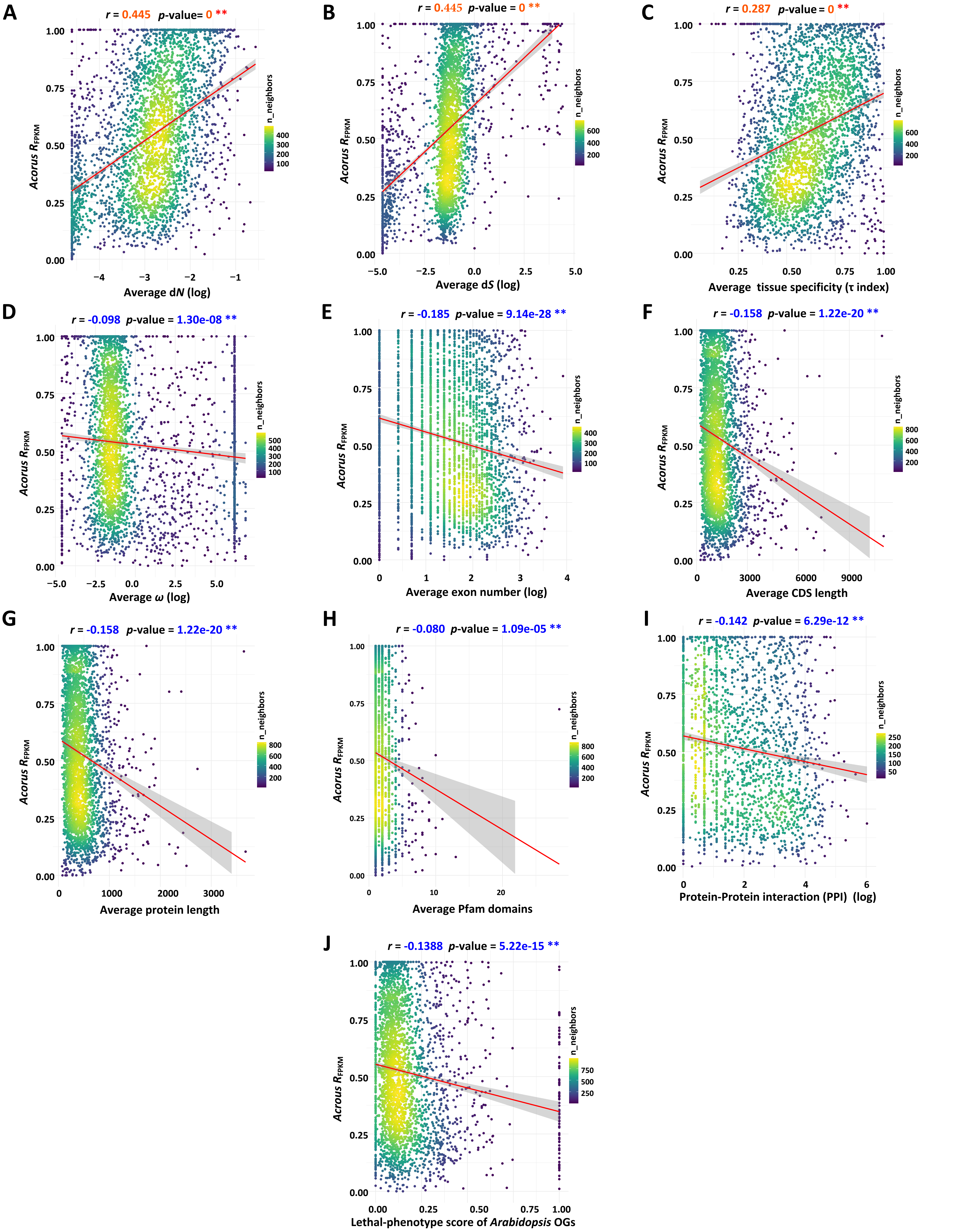

Supplementary Figure S4. Pearson correlations between average relative expression differences (R_{FPKM}) of WGD duplicates and gene features in *Acorus*. A-C. Average R_{FPKM} of WGD duplicates are significantly positively correlated with average non-synonymous substitutions per site (d*N*) after WGD **(A)**, average synonymous substitutions per site (d*S*) after WGD **(B)**, and tissue specificity (τ) of gene expression **(C)**. **D-J.** Average R_{FPKM} of WGD duplicates are significantly negatively correlated with average *ω* (d*N*/d*S* ratio) after WGD **(D),** average exon number **(E)**, average coding sequence (CDS) length **(F)**, average protein length **(G)**, No. of Pfam domains genes **(H)**, No. of protein-protein interactions (PPIs) of putative orthologs in *Arabidopsis* **(I)**, lethal-phenotype score of *Arabidopsis* putative ortholog groups **(J)**. WGD, whole genome duplication; FPKM, fragments per kilobase of exon model per million mapped fragments; *r*, correlation coefficient of Pearson correlation test; log, log-transformed values of gene features in x-axis; grey shading band, the 95% (default) confidence level interval for predictions from a linear model; red font, positive correlation; blue font, negative correlation; *, *p*-value < 0.05; **, *p*-value < 0.01.

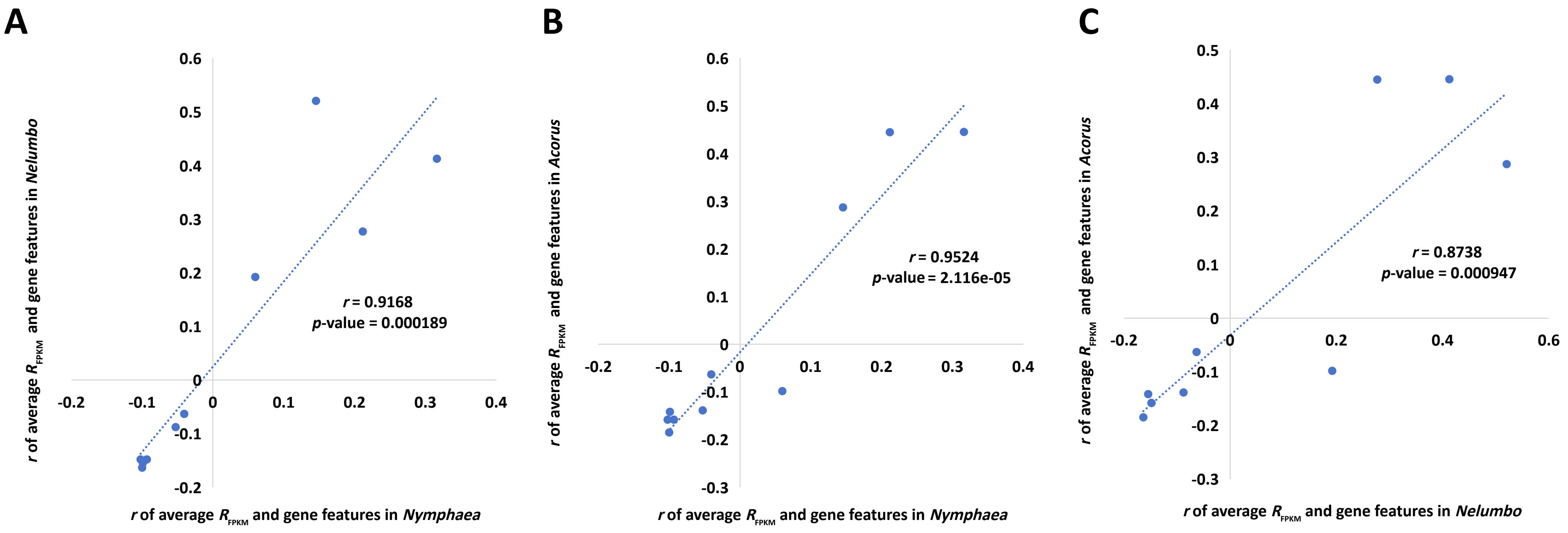

Supplementary Figure S5. Pearson correlations of the correlation coefficients (*r*) of average relative expression (R_{FPKM}) and gene features among three species. (A) *r* of average R_{FPKM} and gene features between *Nymphaea* and *Nelumbo*. **(B)** *r* of average R_{FPKM} and gene features between *Nymphaea* and *Acorus*. **(C)** *r* of average R_{FPKM} and gene features between *Nelumbo* and *Acorus*. FPKM, fragments per kilobase of exon model per million mapped fragments. Gene features were listed in Supplementary Table S6.

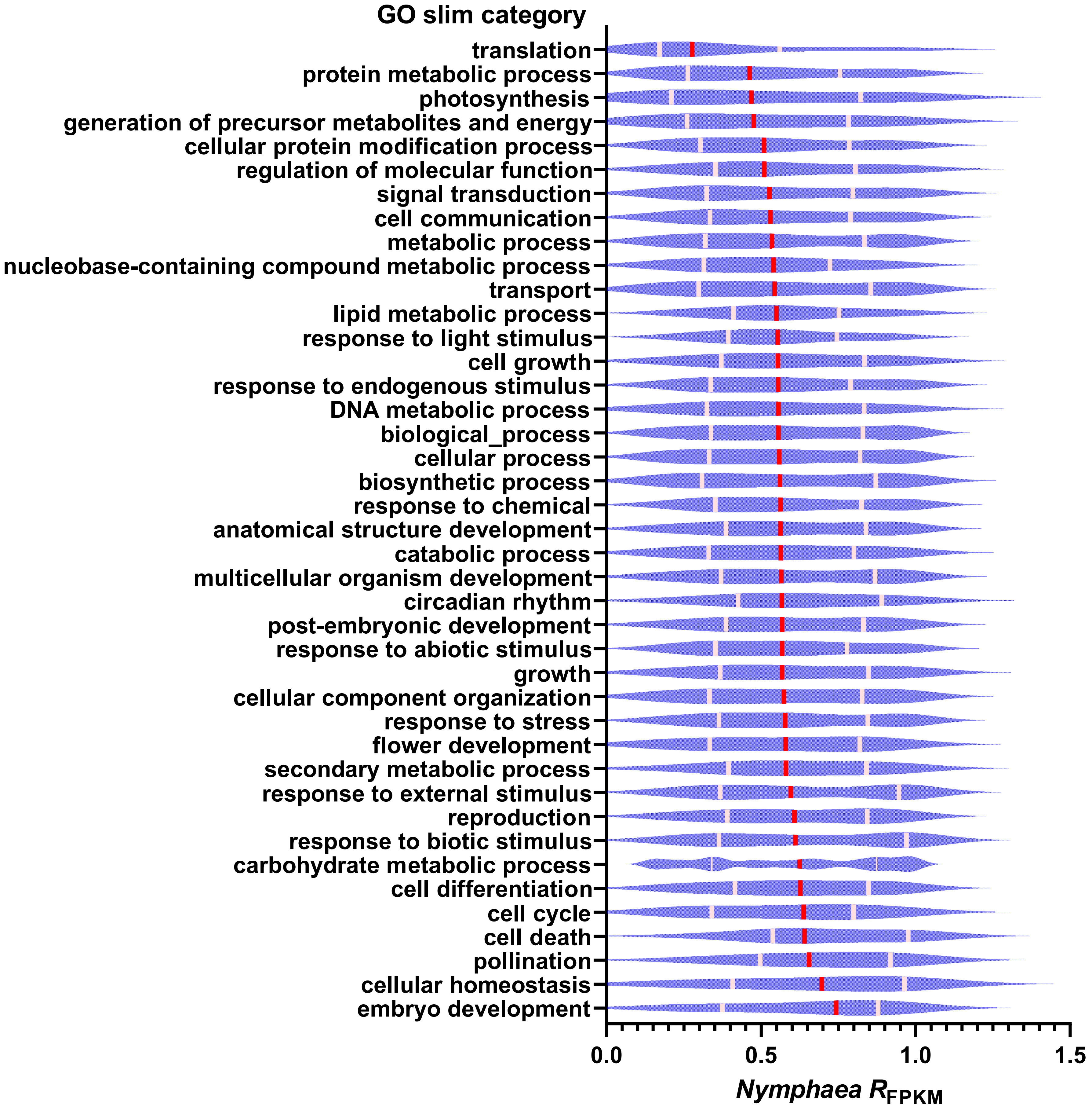

Supplementary Figure S6. Violin plot showing how the relative expression difference (R_{FPKM}) for *Nymphaea* homoeologs varies among duplicate genes belonging to different Gene Ontology (GO) slim categories. FPKM, fragments per kilobase of exon model per million mapped fragments; red bar, median; pink bar, quartile. The sample sizes of the GO slim categories, from bottom to top in the figure, are as follows: 37, 14, 17, 14, 28, 81, 44, 103, 125, 139, 25, 32, 244, 172, 47, 206, 126, 13, 212, 85, 243, 268, 183, 660, 955, 19, 180, 40, 80, 41, 181, 78, 485, 153, 122, 35, 131, 42, 32, 213 and 48.

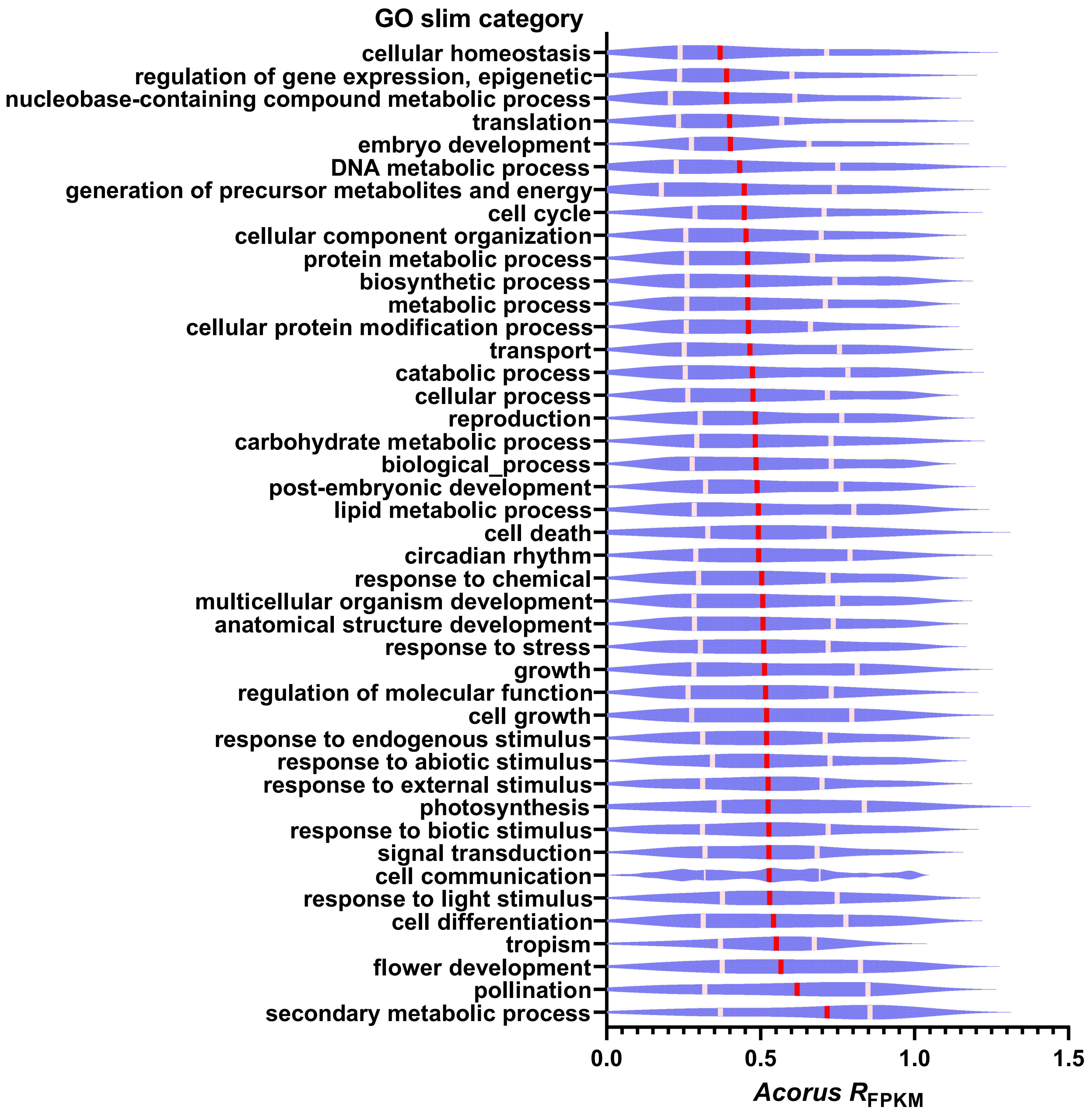

Supplementary Figure S7. Violin plot showing how the relative expression difference (R_{FPKM}) of *Acorus* homoeologs varies among duplicate genes belonging to different Gene Ontology (GO) slim categories. FPKM, fragments per kilobase of exon model per million mapped fragments; red bar, median; pink bar, quartile. The sample sizes of the GO slim categories, from bottom to top in the figure, are as follows: 43, 30, 62, 16, 149, 106, 219, 192, 165, 16, 222, 353, 281, 54, 67, 66, 445, 426, 365, 463, 21, 17, 109, 222, 1559, 72, 218, 1110, 164, 248, 247, 847, 302, 360, 302, 52, 34, 39, 68, 50, 194, 24 and 21.

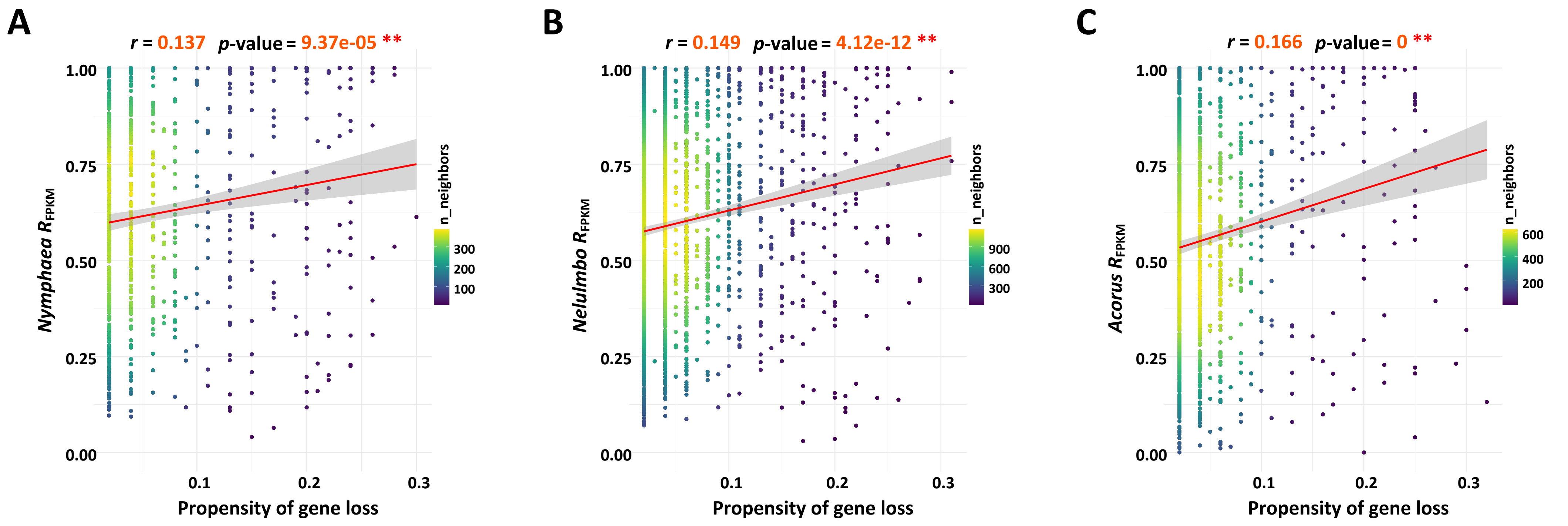

Supplementary Figure S8. Pearson correlations between average relative expression differences (R_{FPKM}) of WGD duplicates and propensity of gene loss. A. *Nymphaea*. **B.** *Nelumbo*. **C.** *Acorus*. WGD, whole genome duplication; FPKM, fragments per kilobase of exon model per million mapped fragments; grey shading band, the 95% (default) confidence level interval for predictions from a linear model; red font, positive correlation; **, p-value < 0.01.

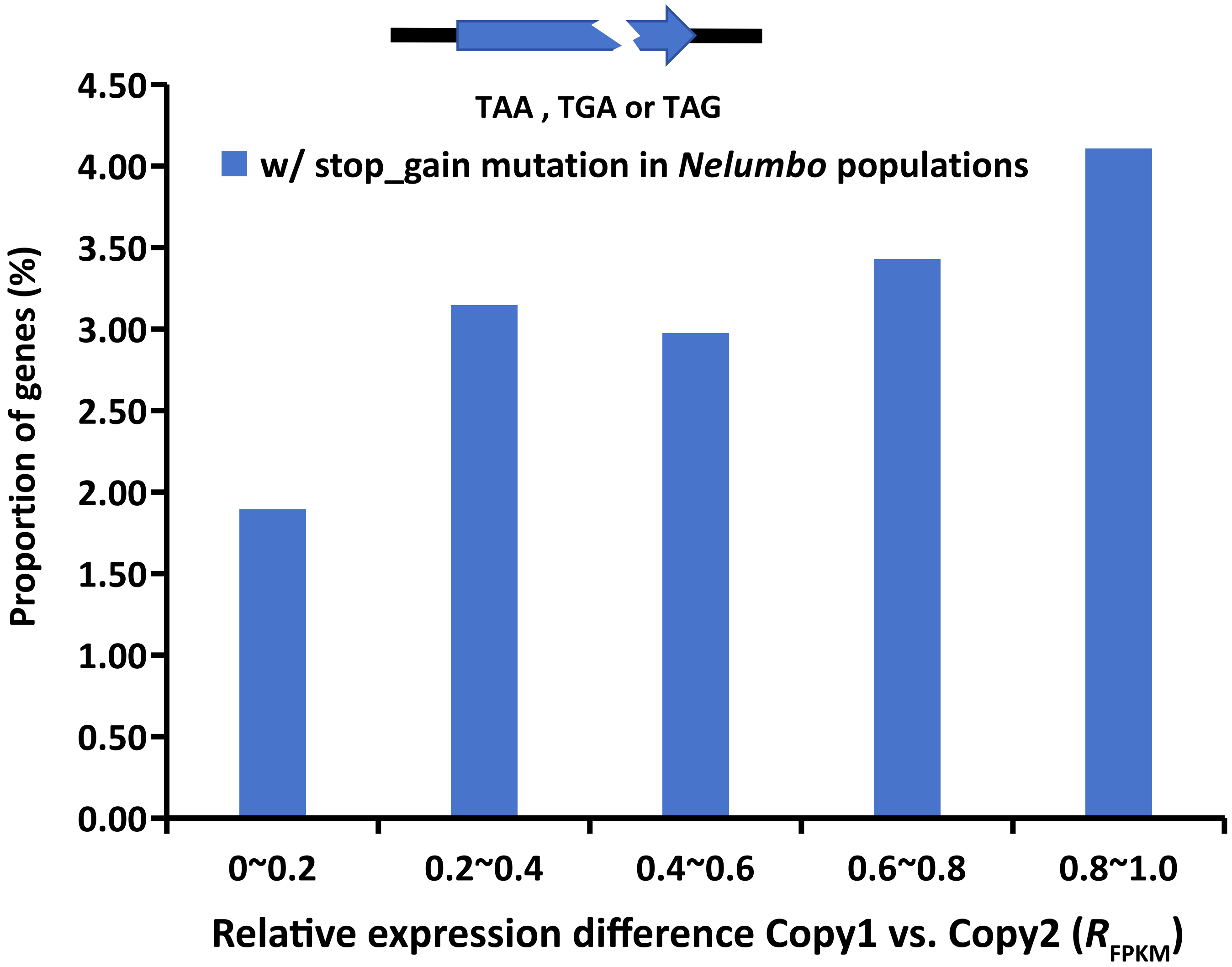

Supplementary Figure S9. Distributions of the proportion of genes with premature stop codon mutations in *Nelumbo* populations according to relative expression difference between the two copies (R_{FPKM}) . FPKM, fragments per kilobase of exon model per million mapped fragments.

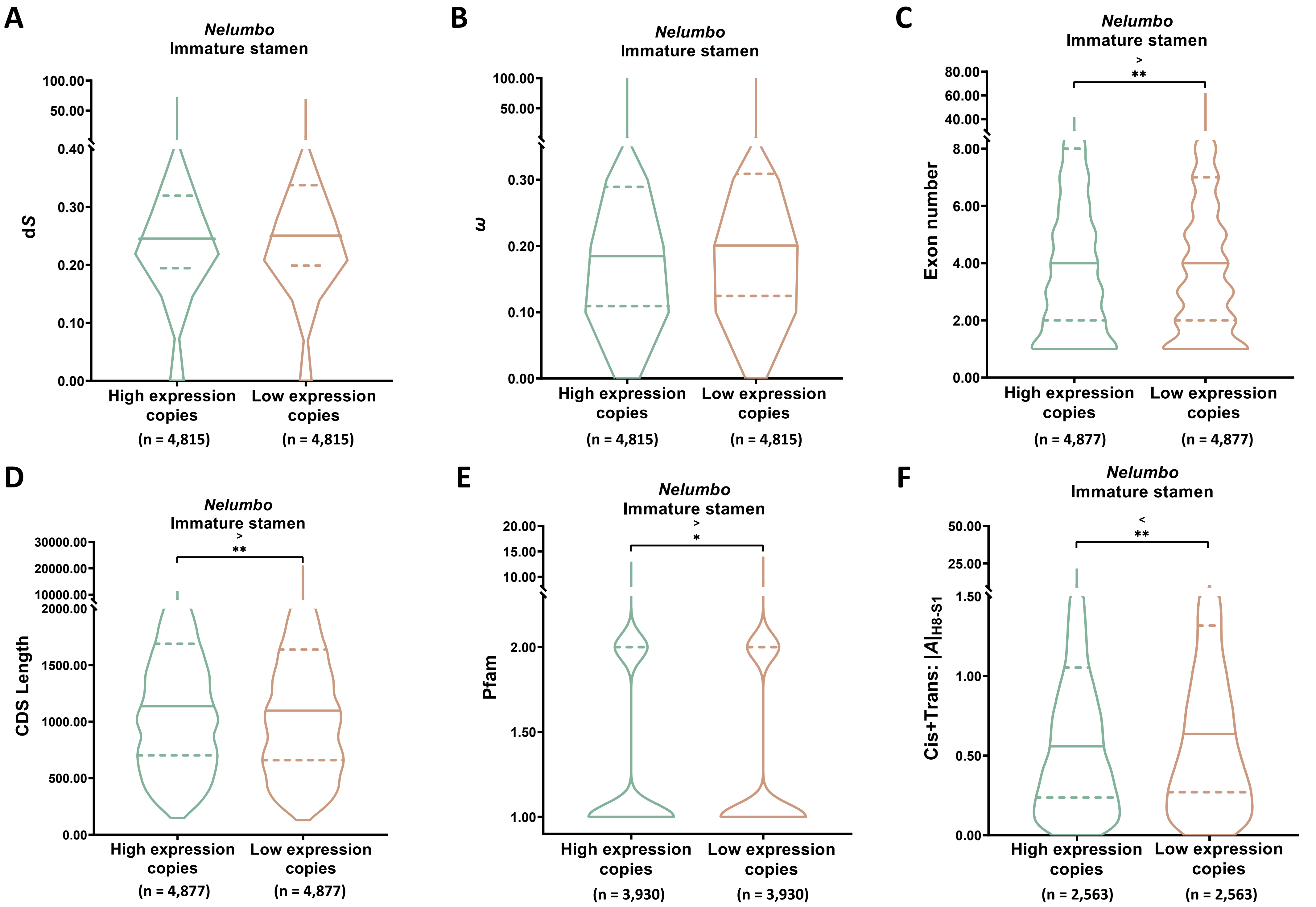

Supplementary Figure S10. Differences between homoeologs with higher and lower gene expression in immature stamen of *Nelumbo*. Differences in synonymous substitutions per site (d*S*) after whole-genome duplication (WGD) **(A)**, *ω* (d*N*/d*S* ratio) after WGD **(B)**, exon number **(C)**, coding sequence (CDS) length **(D)**, No. of Pfam domains of genes **(E)**, *cis*- and *trans*-regulatory change magnitude (H8-S1) **(F)** between homoeologs with higher expression and lower expression in immature stamen of *Nelumbo* (paired *t* tests, ** *p*-value < 0.01, * *p*-value < 0.05). Solid line in violin plot, median value; dashed line in violin plot, quantile; n, sample size.

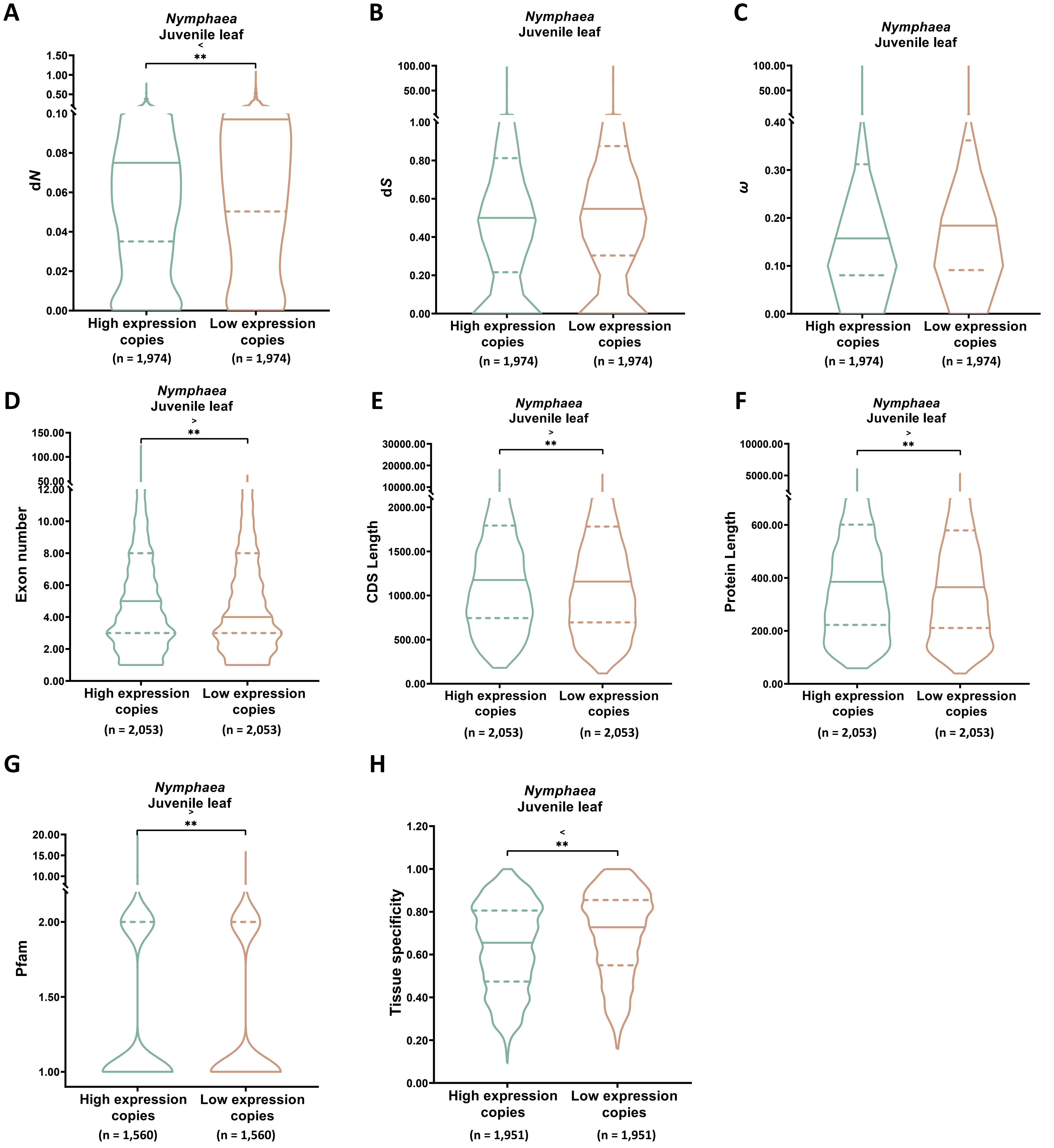

Supplementary Figure S11. Differences between homoeologs with higher and lower gene expression in juvenile leaf of *Nymphaea*. Differences in non-synonymous substitutions per site (d*N*) after whole-genome duplication (WGD) **(A)**, synonymous substitutions per site (d*S*) after WGD **(B)**, *ω* (d*N*/d*S* ratio) after WGD **(C)**, exon number **(D)**, coding sequence (CDS) length **(E)**, protein length **(F)**, No. of Pfam domains of genes **(G)**, tissue specificity (τ index) of gene expression **(H)** between homoeologs with higher expression and lower expression in juvenile leaf of *Nymphaea* (paired *t* tests, ** *p*-value < 0.01)**.** Solid line in violin plot, median value; dashed line in violin plot, quantile; n, sample size.

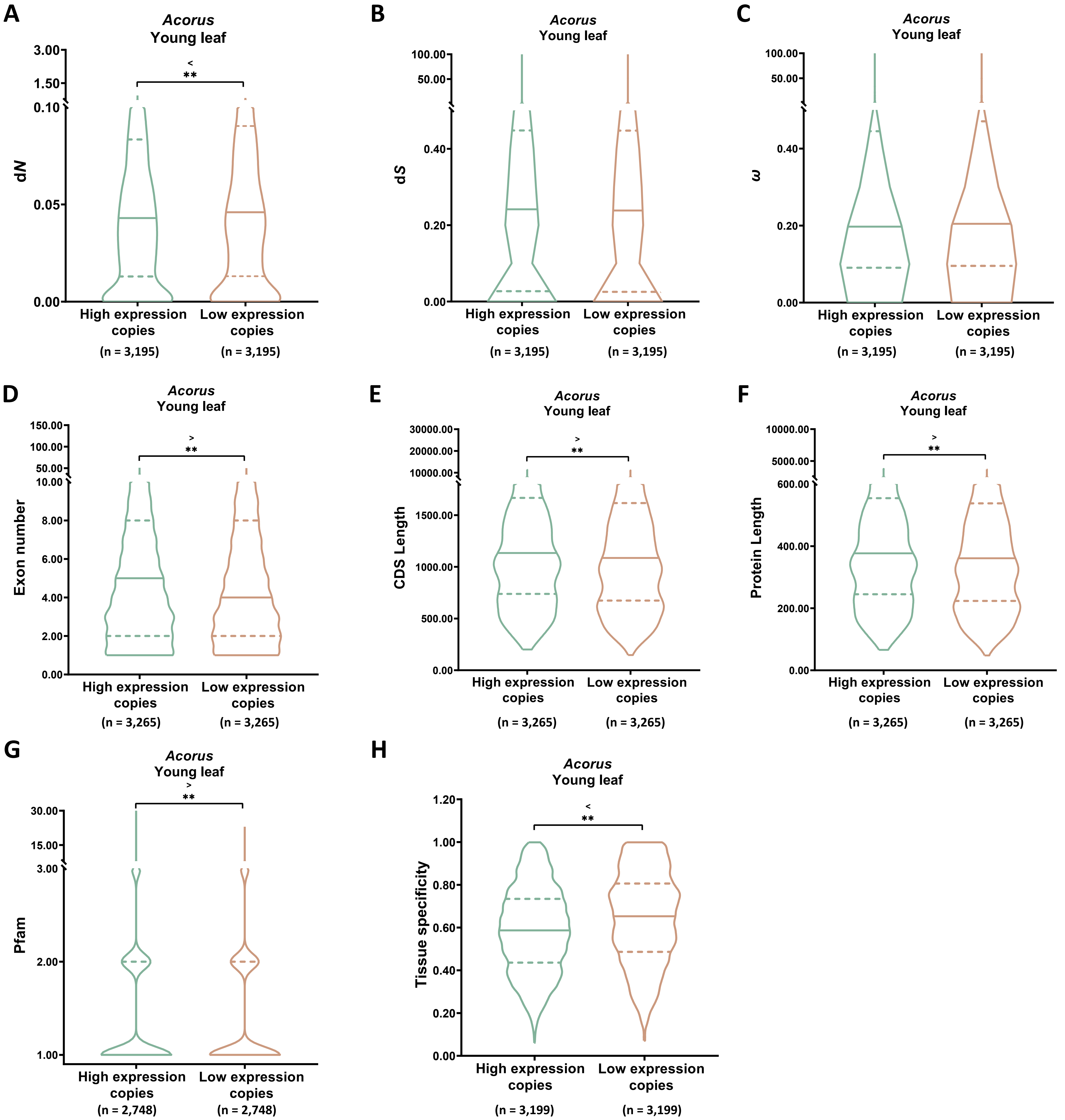

Supplementary Figure S12. Differences between homoeologs with higher and lower gene expression in young leaf of *Acorus*. Differences in non-synonymous substitutions per site (d*N*) after whole-genome duplication (WGD) **(A)**, synonymous substitutions per site (d*S*) after WGD **(B)**, *ω* (d*N*/d*S* ratio) after WGD **(C)**, exon number **(D)**, coding sequence (CDS) length **(E)**, protein length **(F)**, No. of Pfam domains of genes **(G)**, tissue specificity (τ index) of gene expression **(H)** between homoeologs with higher expression and lower expression in young leaf of *Acorus* (paired *t* tests, ** *p*-value < 0.01)**.** Solid line in violin plot, median value; dashed line in violin plot, quantile; n, sample size.

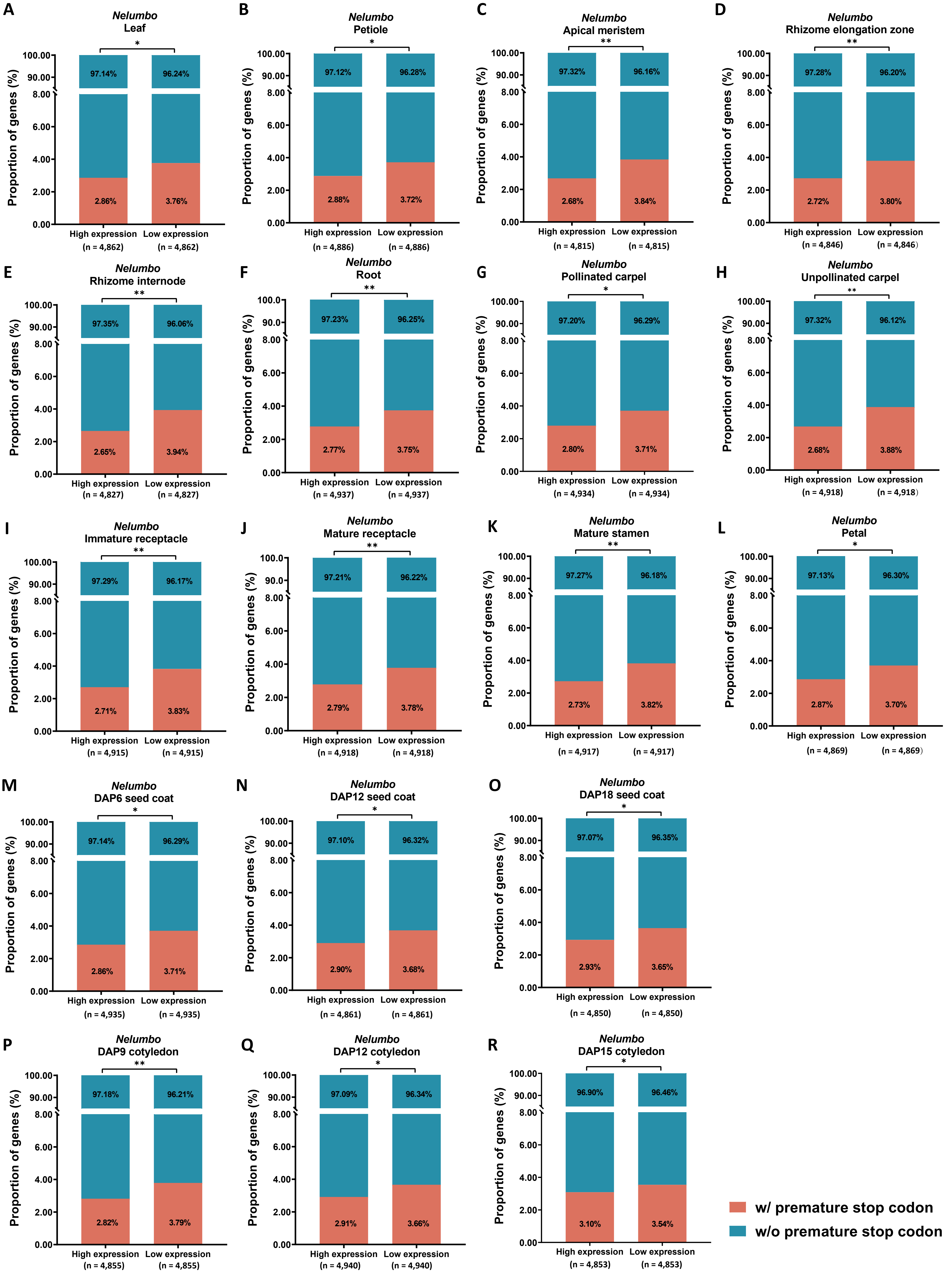

Supplementary Figure S13. Differences in premature stop codon mutations in *Nelumbo* populations between homoeologs with higher expression and lower expression in 19 different tissues of *Nelumbo*. χ^2 test, ** *p*-value < 0.01, * *p*-value < 0.05; n, sample size.
